# Efficacy of high-flow nasal cannula in patients with acute heart failure: a systematic review and meta-analysis

**DOI:** 10.1186/s12890-023-02782-0

**Published:** 2023-11-28

**Authors:** Liming Yan, Ye Lu, Mingming Deng, Qin Zhang, Yiding Bian, Xiaoming Zhou, Gang Hou

**Affiliations:** 1 Department of Pulmonary and Critical Care Medicine, Fourth Hospital of China Medical University, Shenyang, China; 2 Department of Pulmonary and Critical Care Medicine, Shengjing Hospital of China Medical University, Shenyang, China; 3 Department of Pulmonary and Critical Care Medicine, Center of Respiratory Medicine, China-Japan Friendship Hospital, Beijing, China; 4 National Center for Respiratory Medicine, Chinese Academy of Medical Sciences, Beijing, China; 5 Institute of Respiratory Medicine, Chinese Academy of Medical Sciences, Beijing, China; 6 Chinese Academy of Medical Sciences, Peking Union Medical College, Beijing, China; 7 Respiratory Department, Center for Pulmonary Vascular Diseases, Fuwai Hospital, National Center for Cardiovascular Diseases, Chinese Academy of Medical Sciences and Peking Union Medical College, Beijing, People’s Republic of China

**Keywords:** High-flow nasal cannula, Non-invasive mechanical ventilation, Conventional oxygen therapy, Intubation, Acute heart failure

## Abstract

**Background:**

Acute heart failure (AHF) is often associated with diffuse insufficiency and arterial hypoxemia, requiring respiratory support for rapid and effective correction. We aimed to compare the effects of high-flow nasal cannula(HFNC) with those of conventional oxygen therapy(COT) or non-invasive ventilation(NIV) on the prognosis of patients with AHF.

**Methods:**

We performed the search using PubMed, Embase, Web of Science, MEDLINE, the Cochrane Library, CNKI, Wanfang, and VIP databases from the inception to August 31, 2023 for relevant studies in English and Chinese. We included controlled studies comparing HFNC with COT or NIV in patients with AHF. Primary outcomes included the intubation rate, respiratory rate (RR), heart rate (HR), and oxygenation status.

**Results:**

From the 1288 original papers identified, 16 studies met the inclusion criteria, and 1333 patients were included. Compared with COT, HFNC reduced the intubation rate (odds ratio [OR]: 0.29, 95% CI: 0.14–0.58, P = 0.0005), RR (standardized mean difference [SMD]: -0.73 95% CI: -0.99 – -0.47, P < 0.00001) and HR (SMD: -0.88, 95% CI: -1.07 – -0.69, P < 0.00001), and hospital stay (SMD: -0.94, 95% CI: -1.76 – -0.12, P = 0.03), and increase arterial oxygen partial pressure (PaO_2_), (SMD: 0.88, 95% CI: 0.70–1.06, P < 0.00001) and oxygen saturation (SpO_2_ [%], SMD: 0.70, 95% CI: 0.34–1.06, P = 0.0001).

**Conclusions:**

There were no significant differences in intubation rate, RR, HR, arterial blood gas parameters, and dyspnea scores between the HFNC and NIV groups. Compared with COT, HFNC effectively reduced the intubation rate and provided greater clinical benefits to patients with AHF. However, there was no significant difference in the clinical prognosis of patients with AHF between the HFNC and NIV groups.

**Trial registration:**

PROSPERO (identifier: CRD42022365611).

**Supplementary Information:**

The online version contains supplementary material available at 10.1186/s12890-023-02782-0.

## Introduction

Acute heart failure (AHF) is characterized by reduced myocardial contractility and increased cardiac load, caused by acute hemodynamic abnormalities. AHF results in circulatory congestion, often manifested as acute cardiogenic pulmonary edema (ACPE) and cardiogenic shock (CS) commonly companied by significant hypoxemia requiring appropriate respiratory support [1]. If conventional oxygen therapy (COT), such as nasal cannula or facial mask oxygen therapy, fails, respiratory support is escalated to non-invasive ventilation (NIV) and invasive mechanical ventilation in time. NIV is recommended in treatment guidelines for heart failure patients with severe respiratory distress presenting, or those on long-term mechanical ventilation with a risk of extubation failure [2]. However, NIV use may cause discomfort and dryness in the respiratory mucosa, which is more fragile for respiratory bacterial infection [3, 4]. Furthermore, patients may develop facial pressure sores and poor tolerance, leading to treatment failure. High-flow nasal cannula (HFNC), a novel oxygen therapy, provides accurate oxygen concentration and constant temperature and humidity of the high-flow gas as an alternative for patients with acute respiratory distress [5]. High-flow gas delivered by HFNC can produce a positive end-expiratory pressure (PEEP) effect [6], which can improve the diffusion function by increasing alveolar pressure and reducing lung exudation. Therefore, HFNC can prevent alveolar collapse owing to its potential PEEP effect and reduce the intubation rate after extubation in critically ill patients. Furthermore, constant temperature and humidity for comfort improve the tolerance of patients to this type of respiratory support [7]. Several studies have demonstrated that HFNC can improve treatment outcomes for AHF [8–10]. In addition, the safety and efficacy of HFNC in patients with heart failure have also been confirmed [11], providing a promising treatment option for those with acute respiratory failure caused by AHF. However, no definitive conclusion has been reached regarding the benefits of HFNC compared with those of COT or NIV. Thus, this meta-analysis aimed to quantitatively compare the clinical outcomes of HFNC with those of COT or NIV in patients with AHF in recent years. The benefits of HFNC for respiratory support in patients with AHF were quantified by analyzing outcomes such as intubation rate, length of hospital stay, respiratory rate (RR), heart rate (HR), mean arterial pressure (MAP), arterial oxygen partial pressure (PaO_2_), arterial carbon dioxide partial pressure (PaCO_2_), pH, and oxygen saturation (SpO_2_ [%]), to provide evidence for clinical treatment selection in AHF.

## Materials and methods

We performed this systematic review and meta-analysis based on the Handbook of Systematic Review of Cochrane interventions and the guidelines described in the PRISMA statement [12]. The study was registered with the International Prospective Systems Evaluation Register (PROSPERO: CRD42022365611).

### Inclusion criteria

The inclusion criteria were as follows: (1) Study type: randomized controlled trials (RCTs); (2) Subjects: Adult patients with AHF who required oxygen therapy; (3) Intervention: HFNC was used in the experimental group and conventional oxygen or NIV was used in the control group; (4) Outcome measures: at least one of the following results of treatment escalation to invasive ventilation were reported: intubation rate, the length of hospital stays, RR, HR, MAP, PaO_2_, PaCO_2_, pH, SpO_2_ (%), and dyspnea score ( using a visual analog scale ranging from 1 to 10 to assess the severity of dyspnea) [13]; (5) Studies written in English or Chinese.

### Exclusion criteria

The exclusion criteria were as follows: (1) studies that did not involve HFNC, (2) repeated studies, (3) conventional oxygen/NIV was not used in the control group, (4) non-RCT studies, (5) articles that could not provide detailed baseline characteristics, (6) incorrect statistical methods that could not be rectified, (7) studies that could not provide raw data on sample size and results, and (8) studies that are not written in English or Chinese.

### Outcome indicators

The primary outcome measure was the change in objective parameters, including respiratory support escalation to invasive ventilation (quantified as the intubation rate), RR, HR and oxygenation status (quantified as arterial blood gas analysis [ABG] parameters, including PaO_2_, PaCO_2_, and SpO_2_ [%]). Secondary outcomes were MAP, pH, length of hospital stay, and dyspnea score of patients. For studies reporting the outcomes of interest at multiple time points, the longest reported follow-up period was included in the main analysis.

### Literature retrieval strategy

We performed the search using PubMed, Embase, Web of Science, MEDLINE, the Cochrane Library, CNKI, Wanfang, and VIP databases from inception to August 31, 2023, for relevant studies in English and Chinese. We developed a sensitive search strategy based on the concepts of [heart failure], [Non-Invasive Ventilation], [Oxygen therapy] and [Nasal high flow oxygen]. Where available, we used validated search strings and supplemented them with MeSH terms and other controlled vocabularies (shown in Supplementary Appendix 1). In addition, the reference lists of the included studies were checked for eligible eligibility. The detailed searching strategy and results are presented in Supplementary Appendix 1.

### Trial selection

Two researchers (Liming Yan and Ye Lu) first screened the trials independently. During title and abstract screening, duplicated and non-randomized controlled studies were deleted. Then, through full-text retrieval, the studies that met the inclusion criteria were obtained. Disagreements were resolved by discussion with a third researcher (Xiaoming Zhou).

### Data extraction and Quality Assessment

The data extraction strategy is presented in the Supplementary Appendix 1. A standardized data extraction table was used to extract the literature (Supplementary Table 1).

The risk of bias of the included studies was evaluated by two reviewers according to the Cochrane Collaboration Risk of Bias Tool for RCTs (Supplementary Fig. 1). A modified Jadad scoring system [14] was used to evaluate the quality of the studies (1–3: low quality, 4–7: high quality).

### Statistical analysis

The RevMan5.3 statistical software (The Cochrane Collaboration, Copenhagen, Denmark), was used for meta-analysis. Odds ratios (OR) and 95% confidence intervals (CI) were calculated for dichotomous variables, and standardized mean differences (SMD) and 95% CI were calculated for continuous variables. Statistical heterogeneity was assessed with Chi-squared tests, I^2^ statistics, and visualized forest plots. The random effects model was used for data with p≤0.05 and I^2^≥50%, and a subgroup stratified analysis was performed to evaluate the source of heterogeneity.The fixed effect model was used for data with p > 0.05 and I^2^ < 50%. In the analysis, when two-sided p values < 0.05, the results were considered statistically significant.

## Results

### Study selection

A total of 1288 articles were initially retrieved. After title and abstract examination, duplication removal, and screening for inclusion/exclusion criteria, 16 studies with a total of 1333 patients were finally selected. There were 668 patients in the HFNC group, 377 patients in the COT group, and 288 patients in the NIV group. A flowchart of the study selection process is shown in Supplementary Fig. 2, and the characteristics of the included studies are shown in Supplementary Table 1.

### Quality evaluation of the included studies

Due to the uniqueness of the implementation procedure for the intervention and control measures in this study, double-anonymizing the subjects and implementers was challenging; therefore, implementation bias might exist. The risk of bias is shown in Supplementary Fig. 1 and Supplementary Fig. 3. In the sensitivity analysis, for research indicators containing more than 5 articles, we used R 4.4.4 in the sensitivity analysis to exclude each included study, and subsequently, the effect sizes were pooled. After excluding the studies with high heterogeneity, the results remained robust and did not affect the final conclusions. Details of the sensitivity analysis and funnel plots are provided in Supplementary Appendix 2.

### Meta-analysis outcome indicators

#### Intubation

**Fig. 1:**
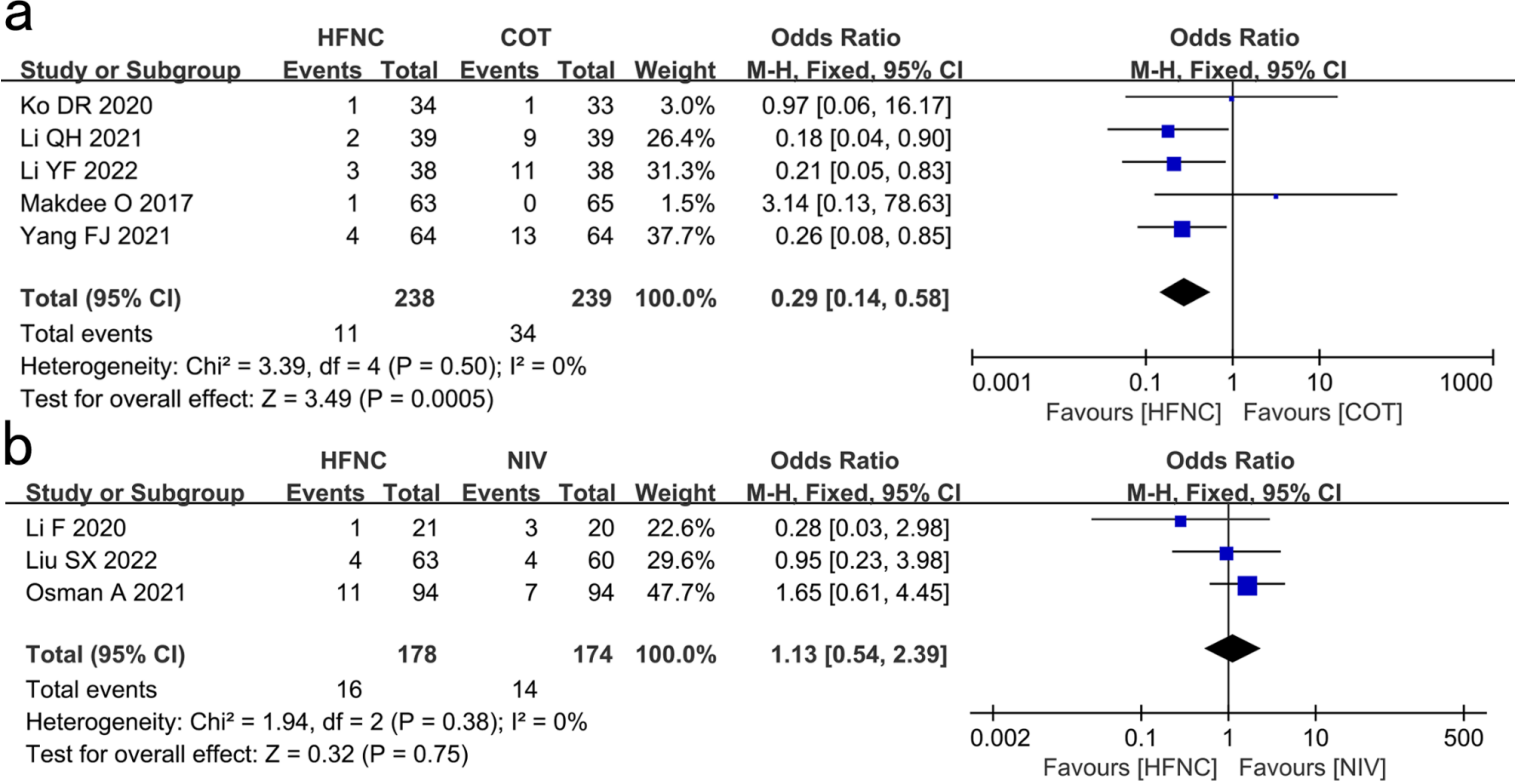
Comparison of intubation rates. **a** High-flow nasal cannula oxygen (HFNC) versus conventional oxygen therapy (COT). **b** HFNC versus noninvasive ventilation (NIV). CI confidence interval, M-H Mantel-Haenszel

Five studies revealed that the intubation rate of the HFNC group was significantly lower than that of the COT group (OR: 0.29, 95% CI: 0.14–0.58, P = 0.0005, I^2^ = 0%; Fig. 1a).

Three studies showed that the intubation rate in the HFNC group was not significantly different from that in the NIV group (OR: 1.13, 95% CI: 0.54–2.39, P = 0.75, I^2^ = 0%; Fig. 1b).

#### RR

**Fig. 2:**
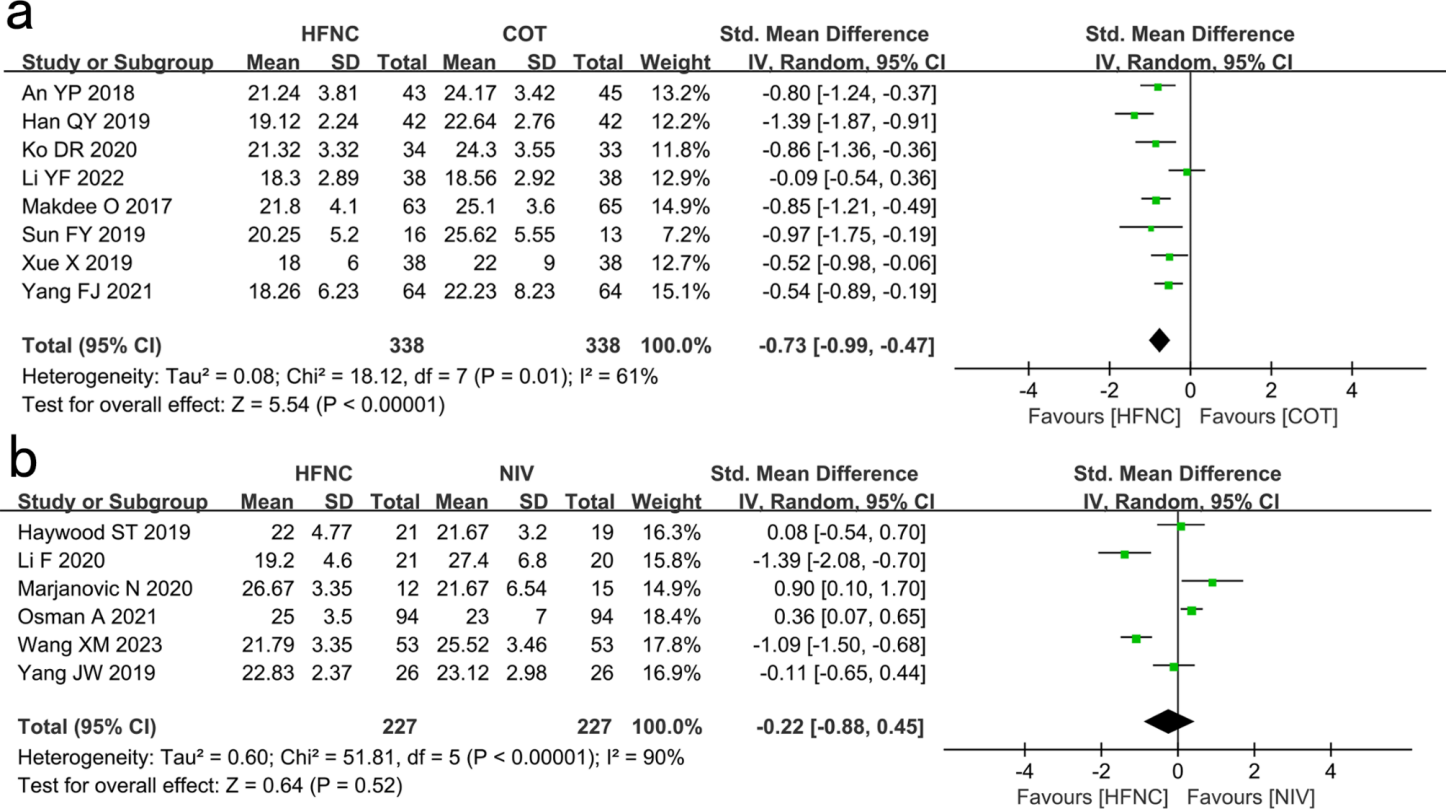
Comparison of RR. **a** High-flow nasal cannula oxygen (HFNC) versus conventional oxygen therapy (COT). **b** HFNC versus noninvasive ventilation (NIV). CI confidence interval, IV Inverse variance

Eight studies showed that RR of the HFNC group was significantly lower than that of the COT group (SMD: -0.73 95% CI: -0.99 – -0.47, P < 0.00001, I^2^ = 61%; Fig. 2a).

Six studies showed that the RR of the HFNC and NIV groups were not significantly different (SMD: -0.22, 95% CI: -0.88–0.45, P = 0.52, I^2^ = 90%; Fig. 2b).

#### PaO_2_

**Fig. 3:**
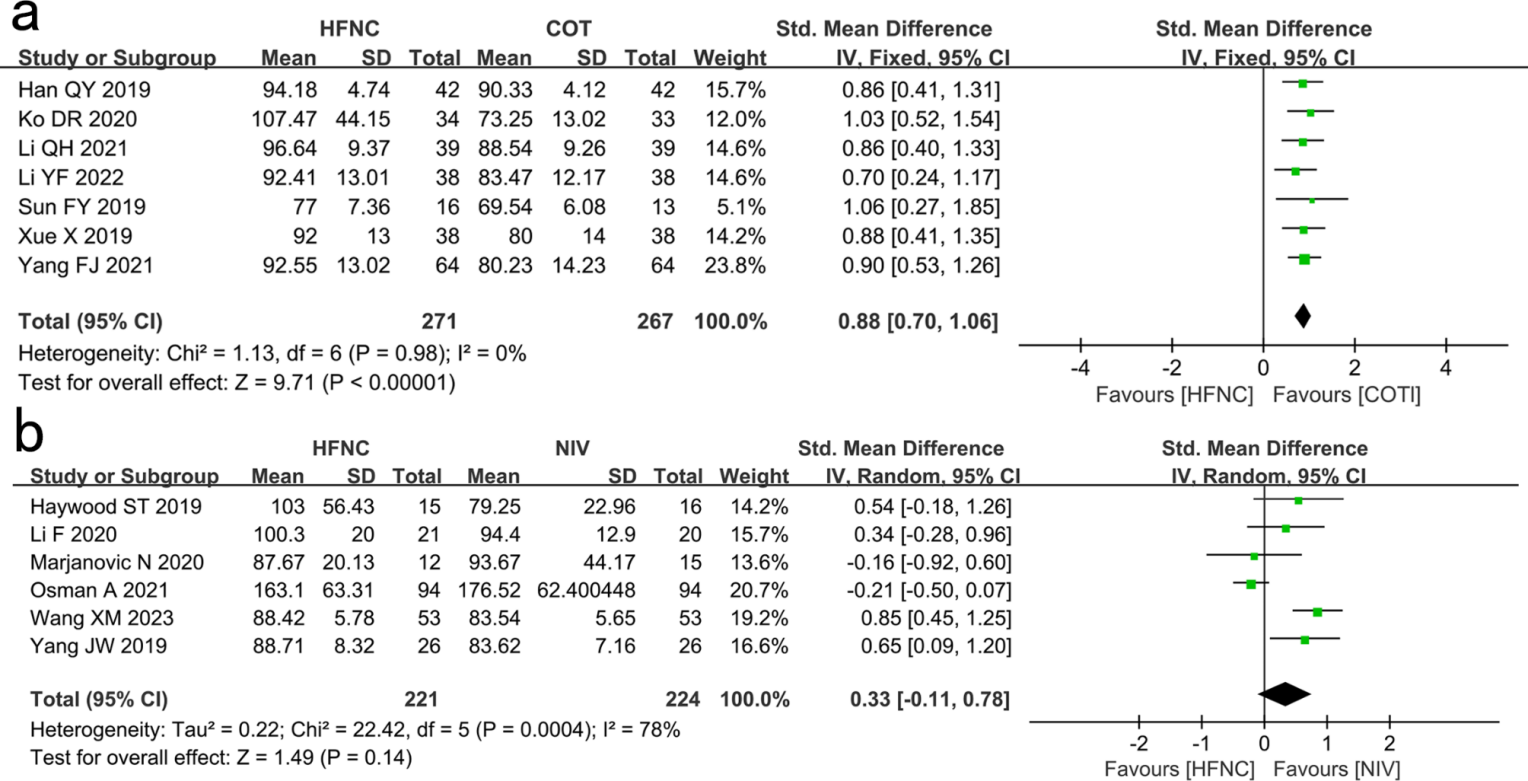
Comparison of PaO_2_. **a** High-flow nasal cannula oxygen (HFNC) versus conventional oxygen therapy (COT). **b** HFNC versus noninvasive ventilation (NIV). CI confidence interval, IV Inverse variance

Seven studies showed that PaO_2_ in the HFNC group was significantly higher than that in the COT group (SMD: 0.88, 95% CI: 0.70–1.06, P < 0.00001, I^2^ = 0%; Fig. 3a).

Six studies showed no significant difference in PaO_2_ between the HFNC and NIV groups. (SMD: 0.33, 95% CI: -0.11–0.78, P = 0.14, I^2^ = 78%; Fig. 3b).

#### PaCO_2_

**Fig. 4:**
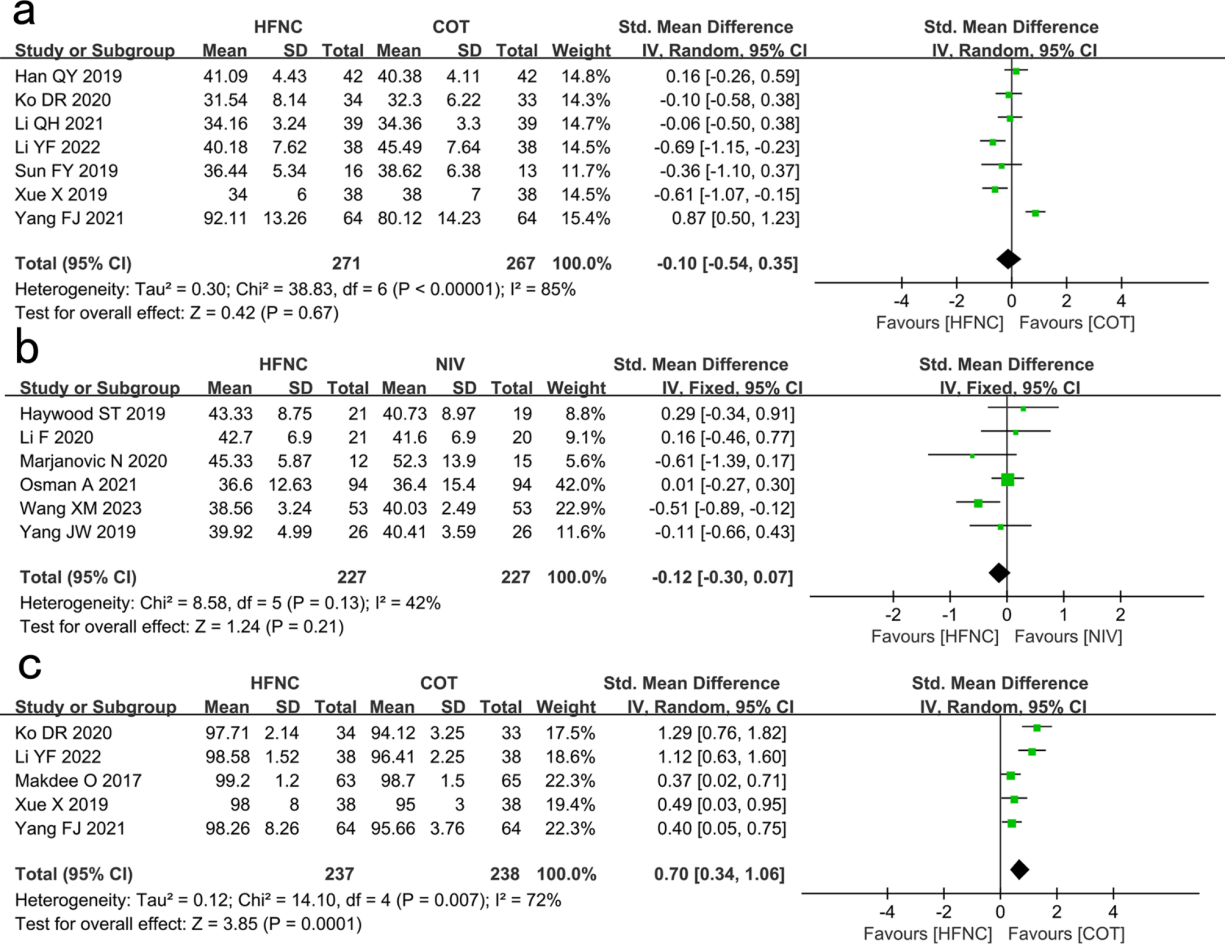
Comparison of PaCO_2_ and SpO_2_ (%). **a** High-flow nasal cannula oxygen (HFNC) versus conventional oxygen therapy (COT). **b** HFNC versus noninvasive ventilation (NIV). **c** Comparison of SpO_2_ (%) in patients who received HFNC compared to COT. CI confidence interval, IV Inverse variance

Seven studies showed no significant difference in PaCO_2_ between the HFNC and COT groups (SMD: -0.10, 95% CI: -0.54–0.35, P = 0.67, I^2^ = 85%; Fig. 4a).

Six studies showed no significant difference in PaCO_2_ between the HFNC and NIV groups (SMD: -0.12, 95% CI: -0.30–0.07, P = 0.21, I^2^ = 42%; Fig. 4b).

#### SpO_2_ (%)

Five studies showed that SpO_2_ in the HFNC group was significantly higher than that in the COT group (SMD: 0.70, 95% CI: 0.34–1.06, P = 0.0001, I^2^ = 72%; Fig. 4c).

#### HR

**Fig. 5:**
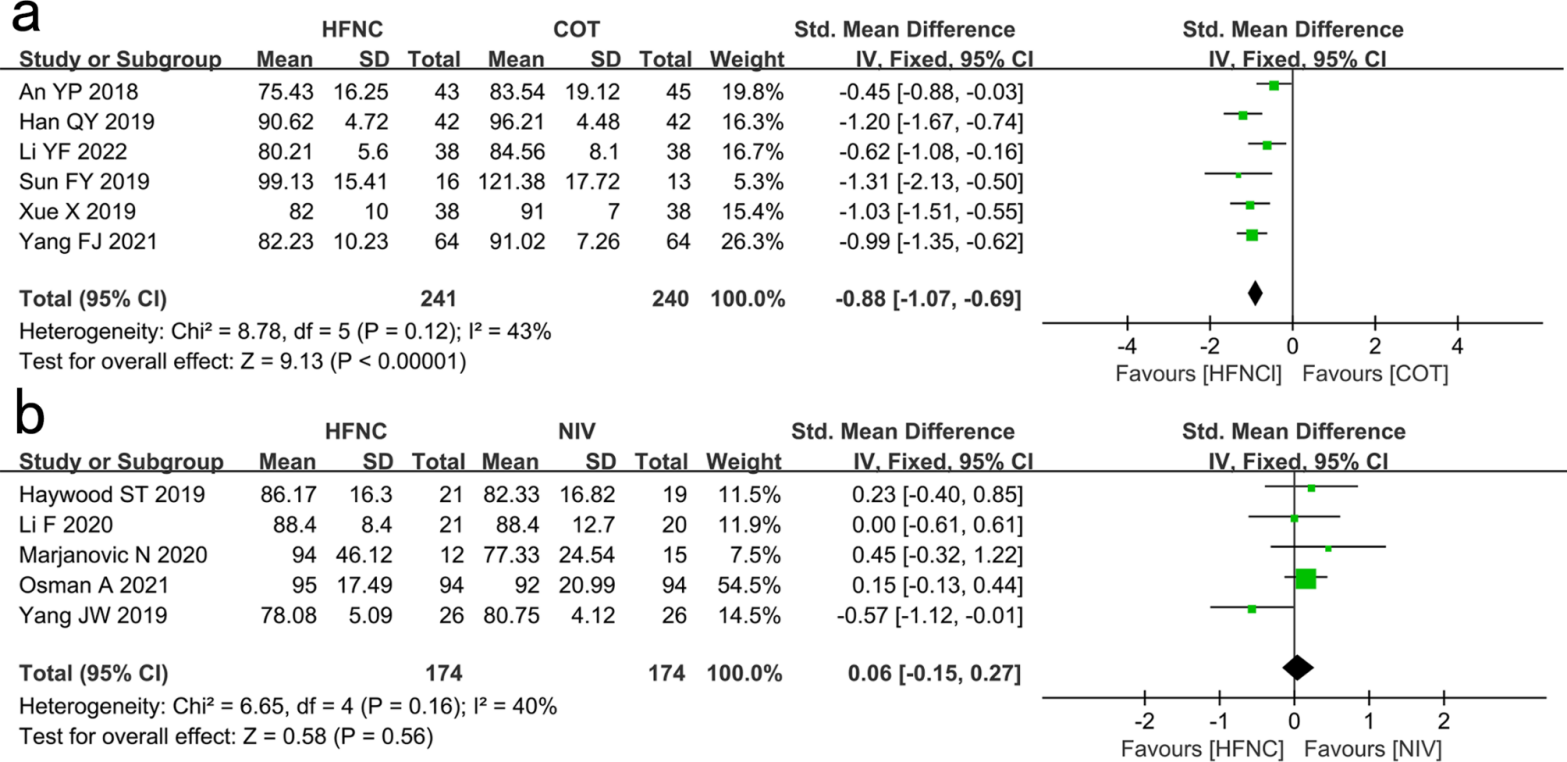
Comparison of HR. **a** High-flow nasal cannula oxygen (HFNC) versus conventional oxygen therapy (COT). **b** HFNC versus noninvasive ventilation (NIV). CI confidence interval, IV Inverse variance

Six studies showed that the HR of the HFNC group was significantly lower than that of the COT group (SMD: -0.88, 95% CI: -1.07 – -0.69, P < 0.00001, I^2^ = 43%; Fig. 5a).

Five studies showed no significant difference in HR between the HFNC and NIV groups (SMD: 0.06, 95% CI: -0.15–0.27, P = 0.56, I^2^ = 40%; Fig. 5b).

#### MAP

Three studies showed no significant difference in MAP between the HFNC and COT groups (SMD: -0.26, 95% CI: -0.87–0.36, P = 0.42, I^2^ = 79%; Supplementary Fig. 4a).

#### Plasma pH

Three studies showed no significant differences in pH between the HFNC and COT groups (SMD: 0.00, 95% CI: -0.29–0.29, P = 1.00, I^2^ = 0%; Supplementary Fig. 4b).

Four studies showed no significant difference in pH between the HFNC and NIV groups (SMD: 0.66, 95% CI: -0.50–1.82, P = 0.27, I^2^ = 94%; Supplementary Fig. 4c).

#### The total length of stay

Four studies showed a significant difference in the total length of hospital stay between the HFNC and the COT groups (SMD: -0.94, 95% CI: -1.76 – -0.12, P = 0.03, I^2^ = 93%; Supplementary Fig. 5a).

#### Dyspnea scores

Four studies showed no significant difference in dyspnea scores between the HFNC and the NIV group (SMD: -1.09, 95% CI: -2.33–0.15, P = 0.08 I^2^ = 96%; Supplementary Fig. 5b).

#### PaO_2_/FiO_2_

Four studies showed no significant difference in PaO_2_/FiO_2_ between the HFNC and the NIV groups (SMD: -0.10, 95% CI: -0.32–0.12, P = 0.38, I^2^ = 0%; Supplementary Fig. 6).

#### Subgroup analyses

Subgroup analysis was performed according to the different end times of the outcomes (1-h treatment vs. 24-h treatment between the HFNC and the COT group; less than 24-h treatment vs. no less than 24-h treatment between the HFNC and the NIV group). Subgroup analysis showed that in the studies comparing HFNC and NIV, the duration of treatment was the source of RR heterogeneity (P-interaction = 0.002, I^2^ = 89.1%; Supplementary Fig. 7), PaO_2_ heterogeneity (P-interaction = 0.008, I^2^ = 85.6%; Supplementary Fig. 8) and HR heterogeneity (P-interaction = 0.04, I^2^ = 76.8%; Supplementary Fig. 9). When comparing HFNC and COT, the subgroup analysis showed that COT had a significant increase in length of stay after 24-hour treatment (P-interaction = 0.0008, I^2^ = 91.1%; Supplementary Fig. 10). However, the other outcomes showed no significant subgroup effects.

Additionally, another subgroup analysis was performed based on the different NIV ventilation models (CPAP, BIPAP and others). Results showed that in the studies comparing HFNC and NIV, different NIV ventilation models was the source of pH heterogeneity (P-interaction < 0.00001, I^2^ = 96.1%; Supplementary Fig. 11) and dyspnea scores heterogeneity (P-interaction < 0.00001, I^2^ = 93.3%; Supplementary Fig. 12). No significant subgroup effects were observed in other outcomes.

Finally, subgroup analysis was conducted in accordance with the various NIV delivery method (helmet or face mask). In the review of the included literature, three studies (Liu SX 2022, Marjanovic N 2020, Haywood ST 2019) mentioned the use of face mask, only one (Osman A 2021) mentioned the use of helmet, and the rest of the studies did not specify how NIV was delivered. According to the analysis by stratification with HFNC versus helmet or versus face mask, the results indicated that different NIV delivery method was the source of RR heterogeneity (P-interaction = 0.008, I^2^ = 79.2%; Supplementary Fig. 13) and pH heterogeneity (P-interaction < 0.00001, I^2^ = 95.7%; Supplementary Fig. 14).

## Discussion

This meta-analysis aimed to evaluate the effects of HFNC, COT, and NIV in adult patients with AHF, highlighting the positive impact of HFNC therapy. Our study primarily demonstrated that compared to COT, HFNC treatment significantly improved the oxygenation status, as indicated by increased PaO_2_ and SpO_2_ levels. Consequently, HFNC therapy reduced the intubation rate and RR in patients with AHF. Additionally, HFNC significantly improved HR, and decreased hospitalization duration with the stabilization of AHF. HFNC produces positive pressure within the nasopharyngeal space, which is appropriate for recruiting collapsed alveoli or increasing the lung volume (PEEP effect) despite its relatively low pressure compared with closed system [15]; this can elevate intrathoracic pressure, decrease venous return, diminish cardiac preload [16], and thus improve the hemodynamic profile of individuals suffering from AHF [17]. Furthermore, this is also the physiological basis for HFNC being better than COT in improving oxygenation status in patients with AHF. HFNC can maintain sufficient oxygenation by improving the respiratory load and gas exchange in cardiogenic pulmonary edema [18]. With a similar PEEP effect, HFNC can benefit patients with NYHA class III chronic heart failure by reducing the collapse of the inferior vena cava and the amount of blood returned to the heart [19]. In addition, HFNC is capable of delivering a substantial flow of oxygen subsequent to the processes of heating and humidification, which has the potential to alleviate the inspiratory force and the work of breathing [20]. Another potential advantage of HFNC over COT is its fast effectiveness, as some studies have suggested that beneficial effects could be achieved by applying HFNC before 30 min [8, 21]. However, in the comparison of HFNC between COT, most of the included studies focused on changes in outcomes from 60-minute to 24-hour. Thus, the subgroup analysis according to the different time dividing line comparing objective indices of respiratory distress (RR, PaO_2_, PaCO_2_ and SpO_2_) showed no significant differences. Based on the findings derived from subgroup analysis, it was not feasible to draw a definitive conclusion on the superiority of the earlier beneficial effects of the application (< 1 h) of HFNC over COT.

In spite of the aforementioned superiority, when compared with COT, HFNC presented no obvious advantage in the changes of PaCO_2_. This result is consistent with the dual role of HFNC in the acute exacerbation of COPD with mild or moderate-to-severe hypercapnia [22]. Setting a flow rate > 40 L/min with HFNC can improve the dynamic lung compliance [23]. Therefore, the use of HFNC can wash out carbon dioxide in anatomical dead space ventilation, resulting in a rapid reduction in PaCO_2_ [24, 25]. However, in a systemic review targeting at the application of HFNC in AECOPD [25], HFNC reduced the work of breathing compared with COT, but keep PaCO_2_ unmodified, while oxygenation slightly deteriorates as opposed to NIV. Dong et al [21] suggested that an improved PaCO_2_ between 0 and 30 min was more effective than COT. However, the difference in PaCO_2_ between the two groups was eliminated 30 min later. The outcome timepoints selected in our study were 1 and 24 h, which might have missed the time when PaCO_2_ was statistically significant. Moreover, AHF often manifested as decreased PO_2_ without hypercapnia; thus, the statistical difference in PCO_2_ was not significant after either HFNC or COT. Future studies are needed to evaluate the optimal time for HFNC to reduce PaCO_2_ in order to maximize the effectiveness of HFNC.

Other outcomes were also explored in some of the included studies. A significant decrease in lactate levels was observed at 2 h in the HFNC group, indicating that HFNC provided more efficient tissue and cell oxygenation [10]. A prospective, randomized, controlled study of patients with acute pulmonary edema demonstrated that HFNC showed better improvement in the lactate clearance rate and objective ABG parameters over time than COT [21]. B-type natriuretic peptide (BNP) and N-terminal pro-BNP have important value in the early detection, risk classification, and prognostic evaluation of heart failure. However, there were too few relevant studies for statistical analysis. One study observed that after 24 h of treatment, N-terminal pro-BNP levels were significantly lower in the HFNC group than in the mask oxygenation group [26].

The use of NIV has been strongly recommended as the initial therapeutic approach for managing acute hypoxia and dyspnea in patients with AHF, as it has the ability to generate significantly higher gas flow rates and positive airway pressure [27, 28]. In our meta-analysis, there was no significant difference in intubation rate, HR, RR, PaO_2_, PaCO_2_, pH, dyspnea score, and PaO_2_ /FiO_2_ between HFNC and NIV. Thus, HFNC therapy could potentially serve as an alternative approach for individuals diagnosed with heart failure. Subgroup analysis was performed according to the different outcome timepoints (≤ 24-h treatment vs. > 24-h treatment between the HFNC and the NIV groups). Subgroup analysis results suggested that NIV reduced RR more significantly when treated for less than 24 h, whereas HFNC reduced RR and increased PaO_2_ more significantly when treated for no less than 24 h. Meanwhile, different treatment timepoints also affected the heterogeneity of heart rate. The different performance of HFNC and NIV based on different timepoints might due to the relatively low and unstable pressure produced by HFNC [15, 16]. HFNC showed its advantages in reducing RR and increasing PaO_2_ with prolonged treatment. Additionally, HFNC therapy exhibits superior patient compliance compared to the intermittent application of NIV [28, 29]. The continuous use of HFNC therapy may guarantee an ample duration of treatment. Based on our preliminary findings, HFNC therapy could potentially serve as an alternative approach for individuals diagnosed with heart failure. However, the specific scope of its application requires further exploration. One study suggested that after 24-hour of HFNC treatment, a PaCO_2_ > 59 mmHg can increase the risk of HFNC failure [30].

Finally, we carried out subgroup analysis based on different NIV ventilation and interface or delivery methods. In our review, three studies (Liu SX 2022 [31], Marjanovic N 2020 [9], Haywood ST 2019 [32]) mentioned the use of face mask, only one (Osman A 2021 [27]) mentioned the use of helmet, and the rest of the studies did not specify how NIV was delivered. Most of the results had no subgroup effect. This could be attributed to the limited number and incomplete information of research included in this analysis. Several of the included studies lacked explicit details regarding NIV delivery methods. Indeed, studies [33–36] from several investigators in the field of acute hypoxic respiratory failure indicate that the type of interface and the method of delivering NIV may influence patient outcomes. In future studies, more information needs to be collected about NIV delivery methods and ventilation patterns to determine which methods lead to better clinical outcomes for patients. Additionally, whether HFNC is appropriate for hypercapnia heart failure is still controversial. Therefore, further studies on the roles of HFNC and NIV in AHF are required.

This analysis has some limitations. First, the methods of oxygen supplementation by COT included in the studies were not uniform, including mask, nasal catheter, nasal obstruction, and venturi mask. Since there are differences in HFNC flow settings and the flow range is wide, it was difficult to conduct subgroup analysis. Second, the sample sizes of some of the included studies were insufficient. The characteristics of the intervention also prevented the researchers from using appropriate blinding, and the quality of the included studies was varied. Additionally, FiO_2_ in the included studies were not recorded in detail, because the titration of oxygenation is dynamic in clinical practice, and the oxygenation target and clinical setting of HFNC or NIV in different studies in different studies varied. Finally, none of the included studies mentioned the effect of delayed intubation on increased mortality, which reflected the neglect of this phenomenon by researchers in the field. We call for future studies to determine the effect of delayed intubation on mortality in patients with heart failure under different oxygen therapy modalities.

## Conclusion

This meta-analysis suggests that compared with COT, HFNC effectively reduced the intubation rate and provided significant clinical benefits to patients with AHF. Additionally, there is no significant difference between HFNC and NIV in the clinical prognosis of AHF, with no definite conclusion regarding the superiority of one to the other. More RCTs with improved design and larger sample sizes are required to evaluate the clinical benefits of HFNC and NIV in patients with AHF, particularly those with different disease severity and underlying comorbidities, to determine the most applicable patient population for HFNC and NIV and to utilize their advantages accordingly.

### Electronic supplementary material

Below is the link to the electronic supplementary material.

**Supplementary Material 1**: **Supplementary Appendix 1**: Detailed search strategies of PubMed, Embase, Web of Science, MEDLINE and the Cochrane Library. **Supplementary Table 1**: Data extraction of the included studies. **Supplementary Fig 1**: Risk of bias of the included studies. **Supplementary Fig 2**. PRISMA flow chart of search and selection of studies

## Data Availability

Raw data were available from the cited articles. In addition, the data generated or analyzed during this study are included in this article and Supplementary Material files.

## Abbreviations

AHF: Acute heart failure
HFNC: high-flow nasal cannula
NIV: non-invasive ventilation
RR: respiratory rate
HR: heart rate
OR: odds ratio
SMD: standardized mean difference
PaO_2_: arterial oxygen partial pressure
PaCO_2_: arterial carbon dioxide partial pressure
SpO_2_: oxygen saturation
MAP: mean arterial pressure
ACPE: acute cardiogenic pulmonary edema
CS: cardiogenic shock
COT: conventional oxygen therapy
PEEP: positive end-expiratory pressure
RCTs: randomized controlled trials
ABG: arterial blood gas
CI: confidence intervals
