# Supplementary material for: Efficacy of high-flow nasal cannula in patients with acute heart failure: a systematic review and meta-analysis
Source: BMC Pulm Med. 2023 Nov 28;23:476. doi: 10.1186/s12890-023-02782-0 (PMC10685599; doi:10.1186/s12890-023-02782-0)

Catalogs

PRISMA checklist······2
Supplementary Table 1-Details of inclued studies······5
Supplementary Figure 1-Overall risk of bias······7
Supplementary Figure 2-Flow diagram of the study selection process······8
Supplementary Figure 3-Methodological quality of trials······9
Supplementary Figure 4-Comparison of MAP and PH······10
Supplementary Figure 5-Comparison of the total length of stay and dyspnea scores······11
Supplementary Figure 6-Comparison of PaO_2/FiO_2······12
Supplementary Figure 7-Subgroup stratified analysis of RR······13
Supplementary Figure 8-Subgroup stratified analysis of PaO_2······14
Supplementary Figure 9-Subgroup stratified analysis of HR······15
Supplementary Figure 10-Subgroup stratified analysis of the total length of stay······16
Supplementary Figure 11-Subgroup stratified analysis of pH ······17
Supplementary Figure 12-Subgroup stratified analysis of dyspnea scores ······18
Supplementary Figure 13-Subgroup stratified analysis of RR ······19
Supplementary Figure 14-Subgroup stratified analysis of pH ······20
Supplementary Appendix 1-Detailed search strategies and extraction strategy······21
Supplementary Appendix 2-Sensitivity analysis and funnel plot······25

PRISMA 2020 Checklist

Section and Topic	Item #	Checklist item	Location where item is reported
**TITLE**			
Title	1	Identify the report as a systematic review.	Page1
**ABSTRACT**			
Abstract	2	See the PRISMA 2020 for Abstracts checklist.	Page1
**INTRODUCTION**			
Rationale	3	Describe the rationale for the review in the context of existing knowledge.	Page1
Objectives	4	Provide an explicit statement of the objective(s) or question(s) the review addresses.	Page2
**METHODS**			
Eligibility criteria	5	Specify the inclusion and exclusion criteria for the review and how studies were grouped for the syntheses.	Page2
Information sources	6	Specify all databases, registers, websites, organisations, reference lists and other sources searched or consulted to identify studies. Specify the date when each source was last searched or consulted.	Page3
Search strategy	7	Present the full search strategies for all databases, registers and websites, including any filters and limits used.	Page3
Selection process	8	Specify the methods used to decide whether a study met the inclusion criteria of the review, including how many reviewers screened each record and each report retrieved, whether they worked independently, and if applicable, details of automation tools used in the process.	Page3
Data collection process	9	Specify the methods used to collect data from reports, including how many reviewers collected data from each report, whether they worked independently, any processes for obtaining or confirming data from study investigators, and if applicable, details of automation tools used in the process.	Page3
Data items	10a	List and define all outcomes for which data were sought. Specify whether all results that were compatible with each outcome domain in each study were sought (e.g. for all measures, time points, analyses), and if not, the methods used to decide which results to collect.	appendix file 1(flow diagram)
	10b	List and define all other variables for which data were sought (e.g. participant and intervention characteristics, funding sources). Describe any assumptions made about any missing or unclear information.	appendix file 1(flow diagram)
Study risk of bias assessment	11	Specify the methods used to assess risk of bias in the included studies, including details of the tool(s) used, how many reviewers assessed each study and whether they worked independently, and if applicable, details of automation tools used in the process.	Page3
Effect measures	12	Specify for each outcome the effect measure(s) (e.g. risk ratio, mean difference) used in the synthesis or presentation of results.	Page4
Synthesis methods	13a	Describe the processes used to decide which studies were eligible for each synthesis (e.g. tabulating the study intervention characteristics and comparing against the planned groups for each synthesis (item #5)).	
	13b	Describe any methods required to prepare the data for presentation or synthesis, such as handling of missing summary statistics, or data conversions.	
	13c	Describe any methods used to tabulate or visually display results of individual studies and syntheses.	
	13d	Describe any methods used to synthesize results and provide a rationale for the choice(s). If meta-analysis was performed, describe the model(s), method(s) to identify the presence and extent of statistical heterogeneity, and software package(s) used.	Page4
	13e	Describe any methods used to explore possible causes of heterogeneity among study results (e.g. subgroup analysis, meta-regression).	
	13f	Describe any sensitivity analyses conducted to assess robustness of the synthesized results.	
Reporting bias assessment	14	Describe any methods used to assess risk of bias due to missing results in a synthesis (arising from reporting biases).	Page3

PRISMA 2020 Checklist

Section and Topic	Item #	Checklist item	Location where item is reported
Certainty assessment	15	Describe any methods used to assess certainty (or confidence) in the body of evidence for an outcome.	Page3
**RESULTS**			
Study selection	16a	Describe the results of the search and selection process, from the number of records identified in the search to the number of studies included in the review, ideally using a flow diagram.	appendix file 1(flow diagram)
	16b	Cite studies that might appear to meet the inclusion criteria, but which were excluded, and explain why they were excluded.	appendix file 1(flow diagram)
Study characteristics	17	Cite each included study and present its characteristics.	appendix file 1(flow diagram)
Risk of bias in studies	18	Present assessments of risk of bias for each included study.	appendix file 2(Risk of bias graph)
Results of individual studies	19	For all outcomes, present, for each study: (a) summary statistics for each group (where appropriate) and (b) an effect estimate and its precision (e.g. confidence/credible interval), ideally using structured tables or plots.	appendix file 1(flow diagram)
Results of syntheses	20a	For each synthesis, briefly summarise the characteristics and risk of bias among contributing studies.	Page6-10
	20b	Present results of all statistical syntheses conducted. If meta-analysis was done, present for each the summary estimate and its precision (e.g. confidence/credible interval) and measures of statistical heterogeneity. If comparing groups, describe the direction of the effect.	Page6-10
	20c	Present results of all investigations of possible causes of heterogeneity among study results.	Page12
	20d	Present results of all sensitivity analyses conducted to assess the robustness of the synthesized results.	Page4
Reporting biases	21	Present assessments of risk of bias due to missing results (arising from reporting biases) for each synthesis assessed.	Page3
Certainty of evidence	22	Present assessments of certainty (or confidence) in the body of evidence for each outcome assessed.	Page6-10
**DISCUSSION**			
Discussion	23a	Provide a general interpretation of the results in the context of other evidence.	Page12
	23b	Discuss any limitations of the evidence included in the review.	Page12
	23c	Discuss any limitations of the review processes used.	Page12
	23d	Discuss implications of the results for practice, policy, and future research.	Page12
**OTHER INFORMATION**			
Registration and protocol	24a	Provide registration information for the review, including register name and registration number, or state that the review was not registered.	Page2
	24b	Indicate where the review protocol can be accessed, or state that a protocol was not prepared.	
	24c	Describe and explain any amendments to information provided at registration or in the protocol.	
Support	25	Describe sources of financial or non-financial support for the review, and the role of the funders or sponsors in the review.	
Competing	26	Declare any competing interests of review authors.	None

Section and Topic	Item #	Checklist item	Location where item is reported
interests			
Availability of data, code and other materials	27	Report which of the following are publicly available and where they can be found: template data collection forms; data extracted from included studies; data used for all analyses; analytic code; any other materials used in the review.	Page3

From: Page MJ, McKenzie JE, Bossuyt PM, Boutron I, Hoffmann TC, Mulrow CD, et al. The PRISMA 2020 statement: an updated guideline for reporting systematic reviews. BMJ 2021;372:n71. doi: 10.1136/bmj.n71

For more information, visit: http://www.prisma-statement.org/

Supplementary Table 1. Details of included studies

Author information	RCT	Number of patients	Sex(M:F)	Average age	general treatment	Sedatiion	Treatment	parameter setting of HFNC group	Baseline RR (times per minute)	Baseline PaO2/FiO2 (mmHg)	Baseline HR (beats per minute)	Baseline PaO2 (mmHg)	Baseline PaCO2 (mmHg)	Baseline SpO2(%)	Baseline LVEF (%)	Control	parameter setting of control group	Baseline RR (times per minute)	Baseline PaO2/FiO2 (mmHg)	Baseline HR (beats per minute)	Baseline PaO2 (mmHg)	Baseline PaCO2 (mmHg)	Baseline SpO2(%)	Baseline LVEF (%)	Outcome indicators	Jadad score
Yang JW 2019[37]	Y	26:26	NA	65.88±12.68/61.92±10.98	Conventional medication for heart failure	NA	HFNC	High flow humidified oxygen therapy system (SpiriOH - 70C), humidified at 37℃, flow rate 40 L/min, FiO2: 50%, adjust the appropriate oxygen concentration to maintain SpO2≥ 92%.	30.86±2.93	228.79±32.49	113.79±7.42	76.04±8.51	39.58±1.61	NA	NA	NIV	S/T(BiPAP), with parameters as follows: IPAP: 10 - 18 cmH20, PEEP ≥ 4 cmH2O, and expiratory tidal volume 6 - 8 ml/kg. The oxygen concentration was gradually adjusted according to SpO2≥ 92%.	31.34±3.20	230.29±30.09	113.29±6.87	75.17±10.60	39.75±2.42	NA	NA	After 2h/6h/24h/48h: RR, HR, PaO_2, $PaCO_2$, PaO2/FiO2; After 48h: tracheal intubation rate, dyspnea, differences in comfort	3
Osman A 2021[27]	Y	94:94	T:C=63:31/60:34	57.3–63.6/58.3–63.4	Standard treatment in accordance with acute heart failure guidelines. No sedative agents.	No	HFNC	Delivered with Hamilton mechanical ventilator, C3S, INTELLiVENT, Switzerland). The gas mixture temperature: 37℃, and absolute humidity: 44mg/L. HFNC was first set at a gas flow of 50 L/min. FiO2 was then titrated to the lowest value compatible with the target >94% SpO2 and maintained throughout the study period.	33(32-25)	152(140-164)	111(108-113)	91.37 (83.88–98.85)	36.62(34.49–38.75)	NA	NA	NIV	Helmet CPAP, the gas flow: 40 L/min, FiO2:0.6, PEEP: 5 cmH2O, titrated upward with increments of 3–5cmH2O (if needed), to achieve oxygen saturation >94%.	35(34–36)	145(138–153)	112(109–115)	87.26(83–92)	36.45(34.21–38.70)	NA	NA	After 1h: RR, HR, PaO_2/FiO_2, HACOR score, dyspnoea scale, and intubation rate	4
Liu SX 2022[31]	Y	63:60	T:C= 39:24/37:23	75.8±14.4/72.9±14.7	Oxygen inhalation, sedation, cardiotonic, diuretic, vasodilator	Sedation was used but no spefic medication recorded	HFNC	The temperature was set at 37 ℃, the initial flow rate was set at 30 L/min, and then titrated up at 5 L/min until the patient felt unwell. The maximum flow rate was 60 L/min, and Oxygen concentration was adjusted to maintain SpO2 > 92%.	19±5	318±95.7	87±17	NA	37.8± 6.1	NA	NA	NIV	BiPAP, through a face mask.Parameters are set from low pressure levels (inspiratory pressure: 6 ~ 8 cm H2O; Expiratory pressure: 4 cm H2O), gradually increased to the appropriate therapeutic level after 10 to 20 min. FiO2 was adjusted to maintain SpO2 > 92%.	20±5	315.7±93.7	89±11	NA	38.9± 9.4	NA	NA	After 48h: intubation rate, length of ICU stay, total length of hospital stay and mortality	3
Li F 2020[38]	Y	21:20	T:C= 9:12/10:10	69.3 ± 7.5/69.9 ± 7.3	Base drugs: digoxin, furosemide, betaloc	NA	HFNC	PSV model; Oxygen flow is 40-60 L/min, FiO2: 40%, and the HFNC oxygen concentration is up or down 10% every 15 min according to the target SpO2.	NA	NA	NA	NA	NA	NA	43. 6 ± 6.8	NIV	PSV, PEEP 5 - 8 cmH2 O, PS: 8 ~ 15 cmH2O FiO2: 40%. Adjust parameters according to target SpO2.	NA	NA	NA	NA	NA	NA	42. 2 ± 7.4	After 1h/6h/24h: pH, PaO_2, PaO_2/FiO_2, $PaCO_2$, RR, MAP, HR; After 24h: dyspnea score, airway humidification score; comfort score	3
Marjanovic N 2020[9]	Y	12:15	T:C= 7:5/3:12	87 (IQR 78–93)	Concomitant standard medical therapy for CPE, including intravenous diuretics and nitrate derivative	NA	HFNC	HFNC was administered via large- or medium-bore binasal prongs, through a face mask or oronasal mask; Flow: 60 L/min, temperature: 37℃, and FIO2 was adjusted to maintain a target SpO2 of at least 92%.	34 (27–41)	NA	97 (80–128)	99 (61–155)	50 (49–61)	93 (90–98)	NA	NIV	NIV was administered through a face mask or oronasal mask; a pressure controlled continuous spontaneous ventilation model; Initial settings were: PEEP 5–10 cmH2O, pressure support 6–10 cm H2O above PEEP, and FIO2 adjusted to a target SpO2 of at least 92%.	29 (26–36)	NA	79 (68–98)	102 (72–125)	60 (48–71)	96 (94–98)	NA	After 1h: $PaCO_2$, pH, breathing frequency, signs of work of breathing and comparisons	3
Haywood ST 2019[32]	Y	22:20	T:C= 5:17/10:10	64.5 (59–77)/ 60 (53–71.5)	NA	NA	HFNC	HVNI was implemented using a small-bore nasal cannula starting at a flow rate of 35LPM at 35-37℃ and FiO2=1.0, and titrated to target.	33 (28–38)	118 (60–166)	93 (84–101)	82 (60–162)	42.5 (32–55)	95.5 (93–100)	NA	NIV	NIPPV was initiated via an oronasal mask, with initial pressures set at IPAP of 10 cmH2O and PEEP: 5 cmH2O, FiO2:1.0, and titrated to target effect.	34 (28–36)	153.5 (81.8–283.5)	106 (92–110)	116.5 (81–283.5)	42 (39–49)	98.5 (94–100)	NA	After 0.5h/1h/1.5h/4h: HR, RR, SpO2 (%), modified Borg score; After 1h/4h: pH, PaO_2, $PaCO_2$; After 4h: PaO2/FiO2	4
Wang XM 2023[39]	Y	53:53	T:C= 29:24/30:23	62.96±5.63/62.94±5.61	Cardiotonic drugs, diuretic drugs, antiasthmatic drugs, vasodilators and other conventional basic treatment.	NA	HFNC	High flow respiratory humidification therapy instrument (Model: PT101AZ), gas flow rate: 40-60 L/min, FiO2: 40%~80%, temperature: 37℃.	38.13±4.64	NA	NA	65.79±6.01	41.98±4.74	NA	NA	NIV	SPN-CPAP model, PEEP: 5-8 cmH2O; PS: 13-19cmH2O; Flow acceleration value 40-60mbar/s; FiO2: 40%. Adjust the noninvasive ventilator and keep the patient's blood oxygen saturation >94%	38.15±4.66	NA	NA	66.26±5.41	42.10±5.41	NA	NA	After 48h: MAP,RR, PaO2, PaCO2, VAS, Borg score,complication	4
Han QY 2019[26]	Y	42:42	T:C= 24:18/25:17	66.2±4.8/65.5±5.4	Upright position, furosemide for diuresis, lanatoside C for cardiac strength, nitroglycerin or sodium nitroprusside for vasodilation, and morphine for sedation. Patients with infection and other predisposing factors were given active anti-infection and primary disease control treatment	Morphine for sedation	HFNC	High flow humidified oxygen therapy system (SpiriOH - 70C), with the optimal temperature (37℃) and 100% relative humidity (44mg/L). FiO2 was titrated according to the SpO2; the SpO2 was maintained at 94%~98%, and dynamic adjustment was made in combination with blood gas analysis.	34.15±3.26	NA	134.16±4.86	70.48±5.27	38.76±4.23	NA	NA	COT	Oxygen is administered using a Venturi mask; specific oxygen flow and concentration are not mentioned.	33.38±3.32	NA	132.24±5.32	71.26±5.63	38.92±3.75	NA	NA	After 2h/6h/24h: HR, RR, PaO2,PaCO2,pH	3
Sun FY 2019[40]	Y	16:13	T:C= 10:6/8:5	66.50±9.93/68.62±9.92	Oxygen inhalation, sedation, cardiotonic, diuretic, vasodilator, correcting electrolyte disturbance and volume balance, relieving spasm and relieving asthma	Sedation was used but no spefic medication recorded	HFNC	Fisher & Paykel's nasal high flow oxygen apparatus and nasal catheters were initially set to flow 45L/min, temperature 37℃, Fi02: 60%-80%, and then gradually increased the flow rate according to oxygenation and symptoms.	39.13±4.72	NA	151.13±21.26	55.31±5.4	33.38±3.59	74.50±6.61	NA	COT	Use conventional nasal catheters, masks and other oxygen therapy methods; specific oxygen flow and concentration are not mentioned.	39.77±4.92	NA	154.31±20.9	57.46±5.32	32.54±3.69	73.54±6.10	NA	After 1h: HR, RR, MAP, BNP, PaO_2, $PaCO_2$, SaO_2, symptom relief time, effective rate.	3
An YP 2018[41]	Y	43:45	T:C= 25:18/24:21	69.47±8.4/71.24±9.11	NA	NA	HFNC	RESPIRCARE High flow oxygen system with FiO2: 40%-50%, initial flow rate of 35L/min, adjustable flow rate (up to 60L/min) and oxygen concentration according to the condition.	31.76±2.8	NA	98.16±24.27	67.2±8.61	NA	92.16±3.14	NA	COT	Oxygen was delivered by a nasal cannula, 5 L/min-10L/min.	30.82±3.5	NA	94.18±20.32	64.23±10.8	NA	93.28±3.98	NA	After 1h: RR, HR, blood pressure, oxygen saturation, partial pressure of oxygen, Brog Score	3
Ko DR 2020[21]	Y	34:33	T:C= 15:19/13:20	77 ± 8/76 ± 9	Standard and concomitant therapy, according to the established treatment guidelines of the AHA for acute pulmonary edema	NA	HFNC	Fisher & Paykel High flow oxygen system, with a flow rate of 45 L/min and FiO2 of 1.0. The FiO2 (from 21% to 100%) and flow rate (up to 60 L/min) in the system were adjusted to maintain an SpO2 of >93%.	28.32 ± 3.86	332.58 ± 70.45	98.32 ± 25.37	69.84 ± 14.79	34.76 ± 13.17	90.31 ± 7.29	NA	COT	Using a conventional nasal cannula at a flow rate of >2 L/min. The flow rate was continuously adjusted to maintain an SpO2 of >93%.	25.18 ± 3.51	342.43±94.17	92.61 ± 19.33	71.91±19.78	30.89 ± 6.18	92.55 ± 3.78	NA	After 0.5h/1h: RR, SpO2 (%), pH, PaO2, PaCO2;After 1h: Lactate; After 24h: the rate of intubation, ICU admission rate; After 28 days: all-cause mortality	2
Makdee O 2017[8]	Y	63:65	T:C= 16:47/29:36	70.4 (15.9)/71.2 (14.2)	Diuretics, nitroglycerin, nebulizer treatments, and oxygen therapy	NA	HFNC	Fisher & Paykel High flow oxygen system with the initial flow rate: 35 L/min, which could be increased to 60 L/min. The FiO2 was adjusted to maintain oxygen saturation ≥95% and was maintained at that level for 60 minutes.	30.8 ± 3.7	NA	84.5 ±18.3	NA	NA	98.3 ± 1.9	NA	COT	Oxygen was delivered by a nasal cannula or nonrebreather mask; specific oxygen flow and concentration are not mentioned.	31.2 ±3.9	NA	87.5 ±23.3	NA	NA	98.2±1.8	NA	After 1h: RR; After 15min/0.5h/1h: SpO_2, pulse rate, blood pressure, dyspnea score; Intubation within 24 h; Mortality in 7 days; rate of adverse events; emergency department and hospital length of stay; pulmonary edema grade	4
Xue X 2019[42]	Y	38:38	21:17/22:16	55 ± 17/ 54 ± 13	Morphine for sedation, rapid diuresis, sodium nitroprusside or nitroglycerin for vasodilation, aminophylline for asthma, digitalis for cardiac strength and other conventional treatment	Morphine for sedation	HFNC	Fisher & Paykel High flow oxygen system, with initial parameter settings: oxygen flow rate: 50 L/min, FiO2: 50%, and temperature: 37 ℃.	26 ± 12	NA	95 ± 19	56 ± 14	40.5 ± 4.5	88 ± 5	46 ± 13	COT	Oxygen was delivered by a nasal cannula; specific oxygen flow and concentration are not mentioned.	28 ± 10	NA	98 ± 23	57 ± 12	41.3 ± 5.5	89 ± 6	46 ± 15	After 2h/6h/12h/24h: PaO2,PaCO2,SaO2,HR,RR,LVEF,SV,NT-proBNP; Intubation rate, 28-day mortality and ICU stay	4
Yang FJ 2021[43]	Y	64:64	T:C= 39:25/38:26	56.66±3.47/56.59±3.53	Morphine for sedation, rapid diuresis, sodium nitroprusside or nitroglycerin for vasodilation, aminophylline for asthma, digitalis for cardiac strength and other conventional treatment	Morphine for sedation	HFNC	Fisher & Paykel High flow oxygen system, with the initial temperature: 37 ℃, FiO2: 40%, the flow rate is set to 40L/min. The oxygen concentration is appropriately adjusted according to the patient's blood gas analysis. When the oxygen saturation of the patient is > 95%, the oxygen concentration is adjusted to 30%.	22.03±9.52	NA	97.26±8.96	70.33±12.93	78.33±13.23	93.03± 6.43	49.23±12.23	COT	Oxygen was delivered by a nasal cannula, 1 -3L/min, and maintain the patient's oxygen concentration between 25% ~ 50%.	23.23±8.56	NA	96.15± 9.03	70.26±13.23	78.23±13.26	93.02±6.23	49.13±12.03	After 2h/ 1 2h/24h: PaO2,PaCO2,SaO2,HR,RR,LVEF,SV,NT-proBNP; Intubation rate, 28-day mortality and ICU stay	4
Li YF 2022[44]	Y	38:38	T:C= 22:16/20:18	56.5±3.5/56.4±3.5	Cardiotonic, diuretic, nitroglycerin and other conventional drug treatment.	NA	HFNC	Fisher & Paykel High flow oxygen system, with the initial temperature: 37 ℃, FiO2: 40%, the flow rate is set to 40L/min. When the oxygen saturation of the patient is > 95%, the oxygen concentration is adjusted to 30%.	21.95±4.41	NA	96.58±8.75	71.23±12.78	72.36±10.31	93.03±5.89	49.23±8.87	COT	Oxygen was delivered by a nasal cannula, with oxygen concentration at 25% ~ 50%, indoor temperature at 18 ~ 22 ℃, humidity at 50% ~ 60%.	22.02±4.32	NA	97.12±8.86	70.86±12.80	72.40±10.28	92.87±5.68	49.15±8.56	After 2 h/ 1 2h/24h: PaO2,PaCO2,SaO2,HR,RR,LVEF,SV,NT-proBNP; Intubation rate, 28-day mortality and hospital length of stay	3
Li QH 2021[45]	Y	39:39	T:C= 22:17/23:16	68.52±16.31/67.28±17.26	Morphine is used for sedation, furosemide or tolasemide diuresis to reduce cardiac preload, nitroglycerin or sodium nitroprusside to dilate blood vessels, and cedilan to strengthen cardiac contractile force	Morphine for sedation	HFNC	Use the high flow oxygen therapy module of the ventilator. The starting parameters were set as follows: oxygen concentration 50%, flow rate 35L/min, temperature 37.5 ℃, oxygen concentration and flow rate were adjusted according to the patient's finger oxygen and respiratory rate.	27.62±11.34	NA	102.24±18.32	55.62±13.2	39.67±4.33	NA	33.22±12.61	COT	Oxygen is administered using a Venturi mask; specific oxygen flow and concentration are not mentioned.	28.24±10.12	NA	104.21±16.67	56.24±11.82	38.82±3.91	NA	32.93±11.52	After 2h/6h/12h/24h: PaO2,PaCO2,LVEF,SV,NT-proBNP; Intubation rate, 28-day mortality , ICU stay, complication	4

RCT randomized controlled trial, *M:F* male/female, *T:C* treatment group/control group, *HFNC* high flow nasal cannula oxygen therapy, *COT* conventional oxygen therapy, *NIV* noninvasive mechanical ventilation, *NR* not reported, *Y* yes, *ICU* intensive care units, *RR* respiratory rate, *HR* heart rate, *MAP* mean arterial pressure, *SaO2* arterial oxygen saturation, *SpO2* oxyhemoglobin saturation, *PaO2/FiO2* oxygenation index, *LVEF* left ventricular ejection fraction, *BNP* brain natriuretic peptide, *EF* ejection fraction, *Lac* serum lactic acid, *HACOR HR* , acidosis, consciousness, Oxygenation and respiratory rate.

[37] Yang J W, Song L P, Han P, et al. Clinical efficacy analysis of high-flow nasal cannula oxygen therapy for acute left heart failure [J]. Beijing Med. 2019, 41(04): 258-262.
[38] Li F, Zheng P, Pan T T, et al. Effect of high-flow nasal cannula oxygen therapy on sequential weaning in patients with acute heart failure [J]. J Clin Internal Medicine, 2020, 37(09): 661-663.
[39] Wang Xingmei. Effect of high-flow nasal cannula oxygen therapy on respiratory and circulatory indexes in patients with acute heart failure in ICU [J]. Chinese and Foreign Med. 2023, 42(21): 87-90+95.
[40] Sun Feiyu, Huang Zhijian. Effect of high-flow nasal cannula oxygen therapy on patients with acute left heart failure complicated with respiratory failure [J]. J Clinical Emergency. 2019, 20(03): 239-242.
[41] Yong-Peng An, Song-ping Luo, Xiu-Zhi Yang. Clinical observation of high-flow nasal cannula oxygen therapy in the treatment of cardiogenic pulmonary edema [J]. Journal of Clinical Emergency Medicine, 2018, 19(11): 762-764.
[42] Xue X, Yang J H, Jia L, et al. Efficacy of high-flow nasal cannula oxygen therapy in patients with acute left heart failure [J]. Chin J Critical Care Med (Electronic edition). 2019, 12(01): 25-30.
[43] Yang F J, GAO J, Wang C P, et al. Effects of high-flow nasal cannula oxygen therapy and conventional oxygen therapy on cardiac function in patients with acute left heart failure [J]. China Coal Industry Med. 2021, 24(04): 409-413.
[44] Li Yingfeng, Jiang Shangxia, Zhang Haizhen. Application of high-flow nasal cannula oxygen therapy in patients with acute left heart failure after extubation of invasive mechanical ventilation [J]. Modern Practical Medicine. 2022, 34(09): 1190-1192.
[45] Li Qiuhong, Zhao Qianwen, Sun Lina. Effect of high-flow nasal cannula oxygen therapy on acute left heart failure [J]. Journal of Clinical Emergency Medicine, 2021, 22(10): 693-696.

Supplementary Figure 1

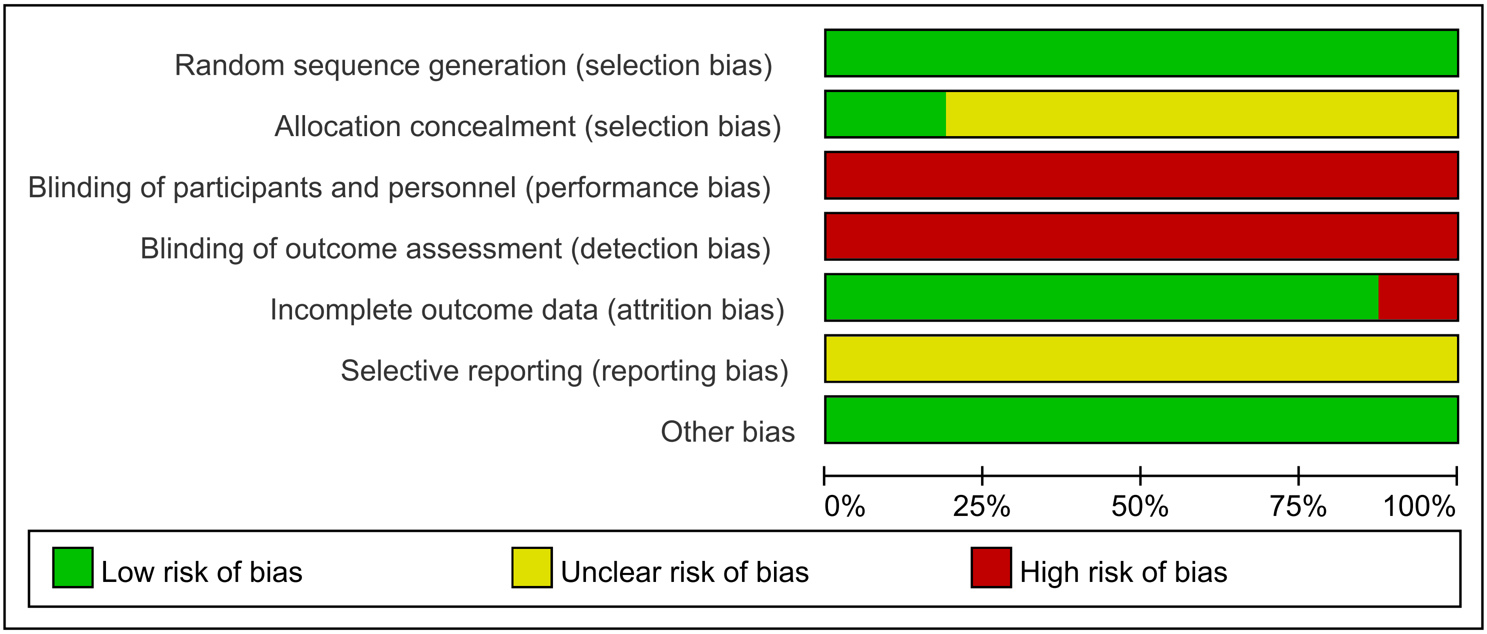

Supplementary Figure 1: Overall risk of bias using the Cochrane risk of bias tool

Supplementary Figure 2 Flow diagram of the study selection process and numbers of studies identified.

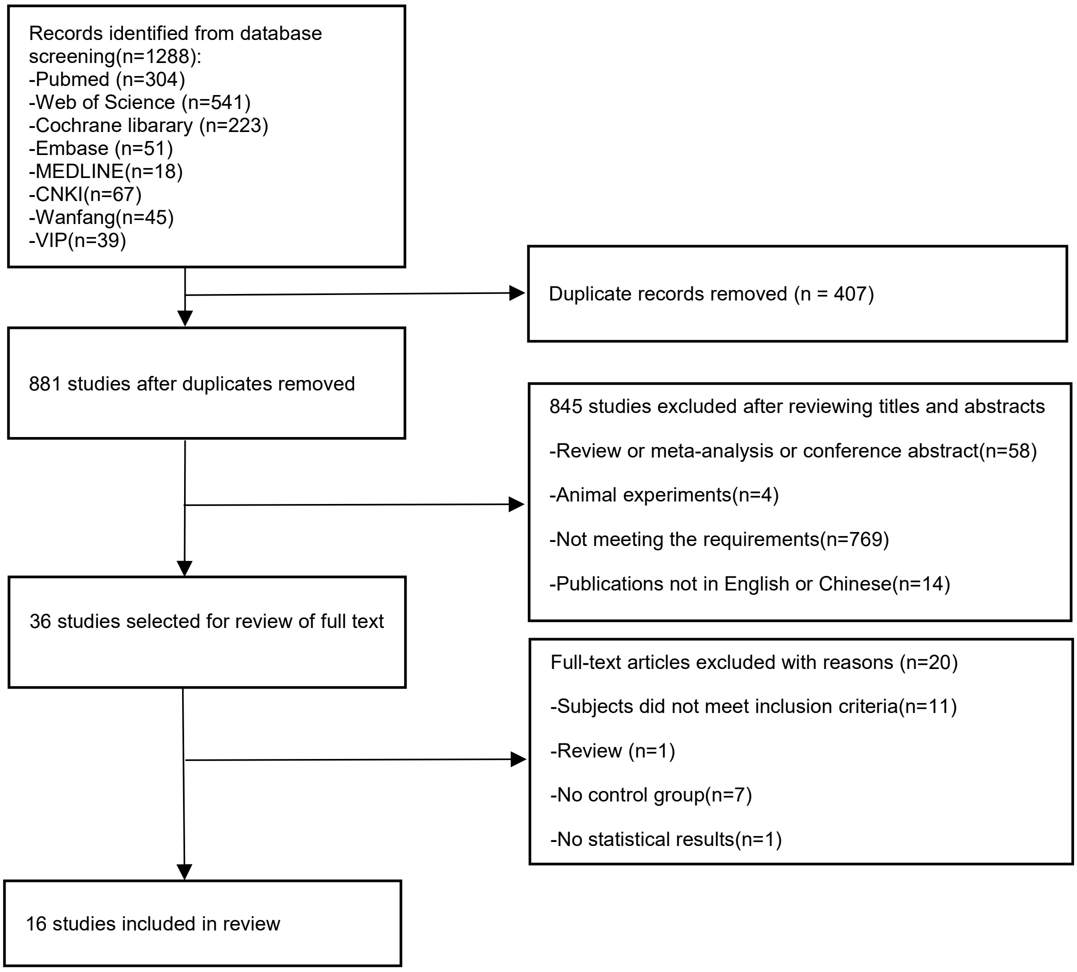

Supplementary Figure 3 Methodological quality of trials.

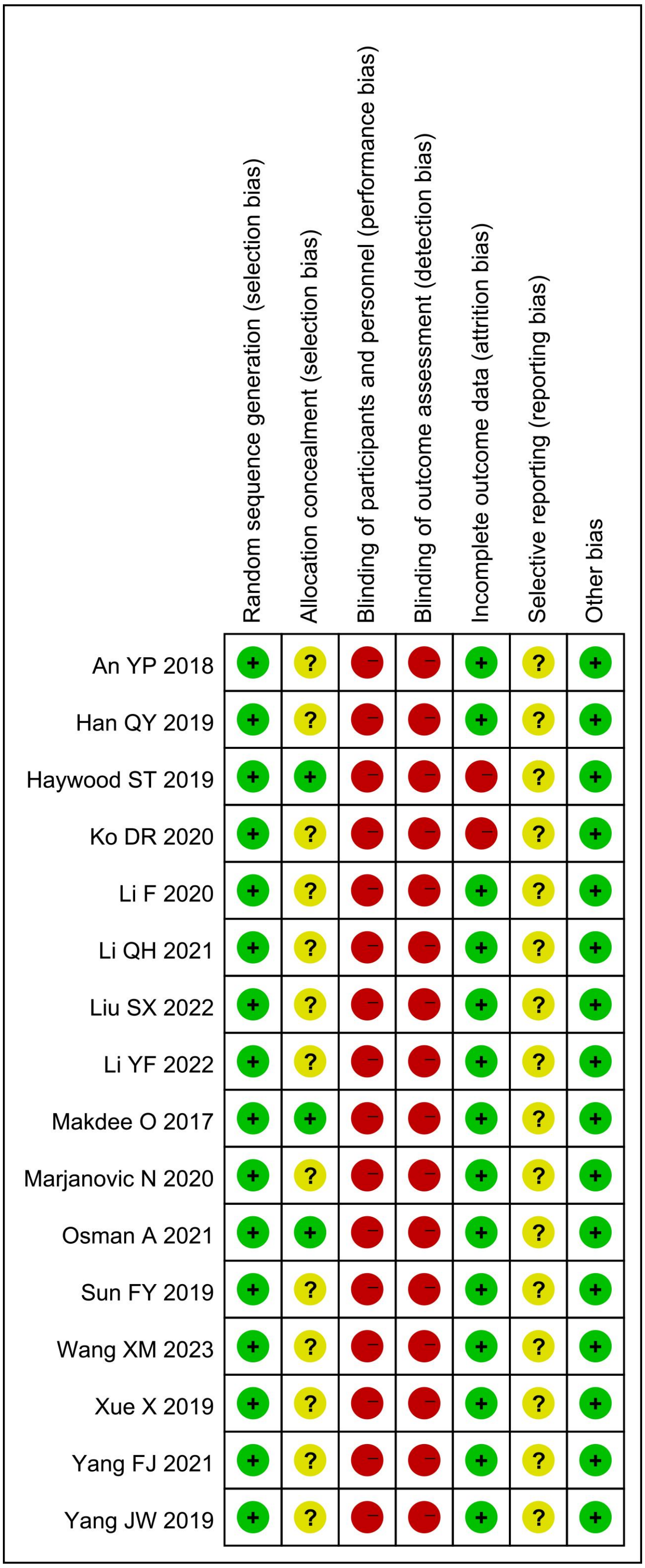

Methodological quality of trials using the Cochrane risk of bias tool. Symbols show low risk of bias (+),unclear risk of bias (?) or high risk of bias (-).

Supplementary Figure 4 Comparison of MAP and PH

a

Study or Subgroup	HFNC Mean	HFNC SD	HFNC Total	COT Mean	COT SD	COT Total	Weight	Std. Mean Difference IV, Random, 95% CI
An YP 2018	81.33	12.71	43	83.92	15.95	45	36.3%	-0.18 [-0.60, 0.24]
Makdee O 2017	94.9	16.9	63	91.3	13.7	65	38.3%	0.23 [-0.11, 0.58]
Sun FY 2019	92.94	13.08	16	110.46	17.84	13	25.3%	-1.11 [-1.90, -0.31]
**Total (95% CI)**			**122**			**123**	**100.0%**	**-0.26 [-0.87, 0.36]**

Heterogeneity: Tau² = 0.23; Chi² = 9.72, df = 2 (P = 0.008); I² = 79%
Test for overall effect: Z = 0.81 (P = 0.42)

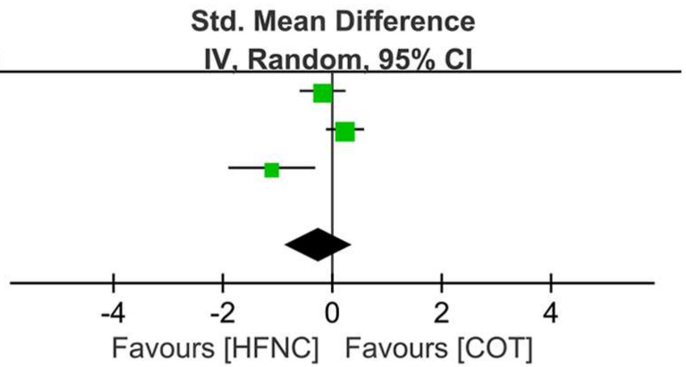

b

Study or Subgroup	HFNC Mean	HFNC SD	HFNC Total	COT Mean	COT SD	COT Total	Weight	Std. Mean Difference IV, Fixed, 95% CI
Han QY 2019	7.41	0.11	42	7.41	0.08	42	46.8%	0.00 [-0.43, 0.43]
Ko DR 2020	7.4	0.06	34	7.4	0.06	33	37.3%	0.00 [-0.48, 0.48]
Sun FY 2019	7.42	0.07	16	7.42	0.06	13	16.0%	0.00 [-0.73, 0.73]
**Total (95% CI)**			**92**			**88**	**100.0%**	**0.00 [-0.29, 0.29]**

Heterogeneity: Chi² = 0.00, df = 2 (P = 1.00); I² = 0%
Test for overall effect: Z = 0.00 (P = 1.00)

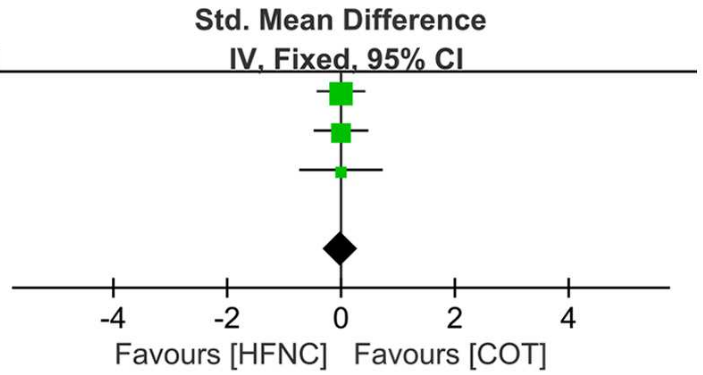

c

Study or Subgroup	HFNC Mean	HFNC SD	HFNC Total	NIV Mean	NIV SD	NIV Total	Weight	Std. Mean Difference IV, Random, 95% CI
Haywood ST 2019	7.38	0.06	21	7.38	0.04	19	24.9%	0.00 [-0.62, 0.62]
Li F 2020	7.4	0.1	21	7.4	0.1	20	25.0%	0.00 [-0.61, 0.61]
Marjanovic N 2020	7.39	0.05	12	7.35	0.08	15	23.9%	0.57 [-0.21, 1.34]
Osman A 2021	7.39	0.01	94	7.37	0.01	94	26.2%	1.99 [1.64, 2.34]
**Total (95% CI)**			**148**			**148**	**100.0%**	**0.66 [-0.50, 1.82]**

Heterogeneity: Tau² = 1.31; Chi² = 51.01, df = 3 (P < 0.00001); I² = 94%
Test for overall effect: Z = 1.11 (P = 0.27)

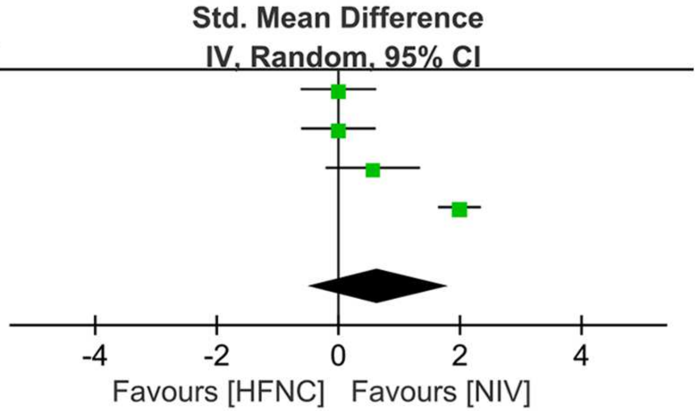

Comparison of MAP and PH. a) Comparison of MAP in patients who received high-flow nasal cannula oxygen (HFNC) compared to conventional oxygen therapy (COT). b) Comparison of PH between HFNC and COT. c) Comparison of pH between HFNC and noninvasive ventilation (NIV). CI confidence interval, IV Inverse variance

Supplementary Figure 5 Comparison of the total length of stay and dyspnea scores

a

Study or Subgroup	HFNC Mean	HFNC SD	HFNC Total	COT Mean	COT SD	COT Total	Weight	Std. Mean Difference IV, Random, 95% CI
Li QH 2021	6.32	2.86	39	9.81	3.12	39	24.6%	-1.15 [-1.64, -0.67]
Li YF 2022	10.42	2.01	38	12.49	3.22	38	24.7%	-0.76 [-1.23, -0.30]
Makdee O 2017	1.1	6.875	63	1.2	4.325	65	25.6%	-0.02 [-0.36, 0.33]
Yang FJ 2021	10.23	2.02	64	15.23	3.26	64	25.1%	-1.83 [-2.25, -1.42]
**Total (95% CI)**			**204**			**206**	**100.0%**	**-0.94 [-1.76, -0.12]**

Heterogeneity: Tau² = 0.65; Chi² = 45.40, df = 3 (P < 0.00001); I² = 93%

Test for overall effect: Z = 2.24 (P = 0.03)

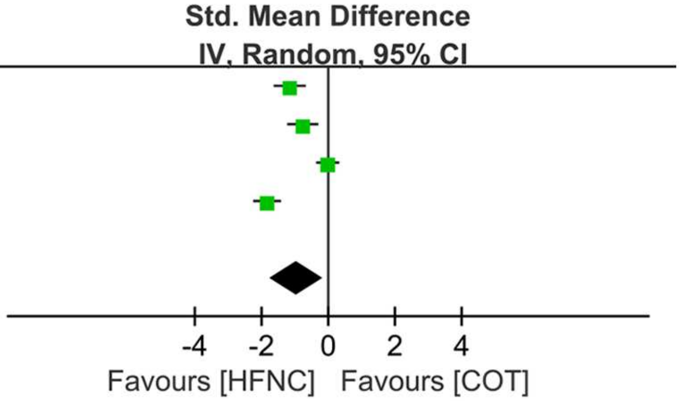

b

Study or Subgroup	HFNC Mean	HFNC SD	HFNC Total	NIV Mean	NIV SD	NIV Total	Weight	Std. Mean Difference IV, Random, 95% CI
Li F 2020	2.5	0.9	21	2.2	0.9	20	24.7%	0.33 [-0.29, 0.94]
Osman A 2021	2.33	3.76	94	3.33	4.52	94	25.9%	-0.24 [-0.53, 0.05]
Wang XM 2023	2.87	0.36	53	3.66	0.35	53	25.2%	-2.21 [-2.70, -1.72]
Yang JW 2019	3.02	0.87	26	4.88	0.72	26	24.2%	-2.29 [-3.01, -1.58]
**Total (95% CI)**			**194**			**193**	**100.0%**	**-1.09 [-2.33, 0.15]**

Heterogeneity: Tau² = 1.52; Chi² = 76.50, df = 3 (P < 0.00001); I² = 96%

Test for overall effect: Z = 1.73 (P = 0.08)

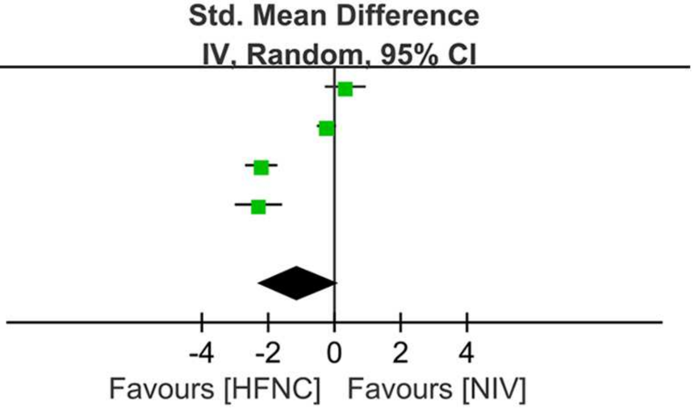

Comparison of the total length of stay and dyspnea scores. a) Comparison of the total length of stay of patients who received high-flow nasal cannula oxygen (HFNC) compared to conventional oxygen therapy (COT). b) Comparison of dyspnea scores of patients who received HFNC compared to noninvasive ventilation (NIV). CI confidence interval, IV Inverse variance

Supplementary Figure 6-Comparison of PaO_2/FiO_2

	HFNC			NIV				Std. Mean Difference
Study or Subgroup	Mean	SD	Total	Mean	SD	Total	Weight	IV, Fixed, 95% CI
Haywood ST 2019	188.9	125.38	15	211.5	92.17	16	9.9%	-0.20 [-0.91, 0.51]
Li F 2020	234.8	50.4	21	225.1	33.9	20	13.1%	0.22 [-0.39, 0.83]
Osman A 2021	272	97.94	94	294	104.93	94	60.2%	-0.22 [-0.50, 0.07]
Yang JW 2019	268.25	43.17	26	263.5	32.48	26	16.7%	0.12 [-0.42, 0.67]
**Total (95% CI)**			**156**			**156**	**100.0%**	**-0.10 [-0.32, 0.12]**

Heterogeneity: $Chi^2 = 2.39$, df = 3 (P = 0.49); $I^2 = 0\%$

Test for overall effect: Z = 0.89 (P = 0.38)

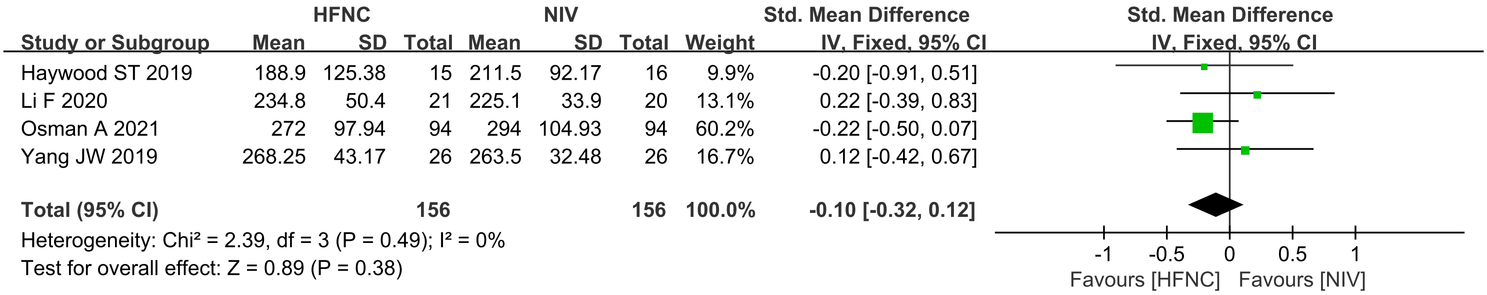

Comparison of PaO2/FiO2 of patients who received high-flow nasal cannula oxygen (HFNC) compared to noninvasive ventilation (NIV). CI confidence interval, IV Inverse variance

Supplementary Figure 7-Subgroup stratified analysis of RR

Study or Subgroup	HFNC Mean	HFNC SD	HFNC Total	NIV Mean	NIV SD	NIV Total	Weight	Std. Mean Difference IV, Random, 95% CI
**1.2.1 less than 24-h treatment**								
Haywood ST 2019	22	4.77	21	21.67	3.2	19	16.3%	0.08 [-0.54, 0.70]
Marjanovic N 2020	26.67	3.35	12	21.67	6.54	15	14.9%	0.90 [0.10, 1.70]
Osman A 2021	25	3.5	94	23	7	94	18.4%	0.36 [0.07, 0.65]
**Subtotal (95% CI)**			**127**			**128**	**49.6%**	**0.37 [0.05, 0.70]**
Heterogeneity: Tau² = 0.02; Chi² = 2.54, df = 2 (P = 0.28); I² = 21%								
Test for overall effect: Z = 2.28 (P = 0.02)								
**1.2.2 no less than 24-h treatment**								
Li F 2020	19.2	4.6	21	27.4	6.8	20	15.8%	-1.39 [-2.08, -0.70]
Wang XM 2023	21.79	3.35	53	25.52	3.46	53	17.8%	-1.09 [-1.50, -0.68]
Yang JW 2019	22.83	2.37	26	23.12	2.98	26	16.9%	-0.11 [-0.65, 0.44]
**Subtotal (95% CI)**			**100**			**99**	**50.4%**	**-0.85 [-1.57, -0.13]**
Heterogeneity: Tau² = 0.33; Chi² = 10.85, df = 2 (P = 0.004); I² = 82%								
Test for overall effect: Z = 2.30 (P = 0.02)								
**Total (95% CI)**			**227**			**227**	**100.0%**	**-0.22 [-0.88, 0.45]**
Heterogeneity: Tau² = 0.60; Chi² = 51.81, df = 5 (P < 0.00001); I² = 90%								
Test for overall effect: Z = 0.64 (P = 0.52)								
Test for subgroup differences: Chi² = 9.18, df = 1 (P = 0.002), I² = 89.1%								

Std. Mean Difference
IV, Random, 95% CI

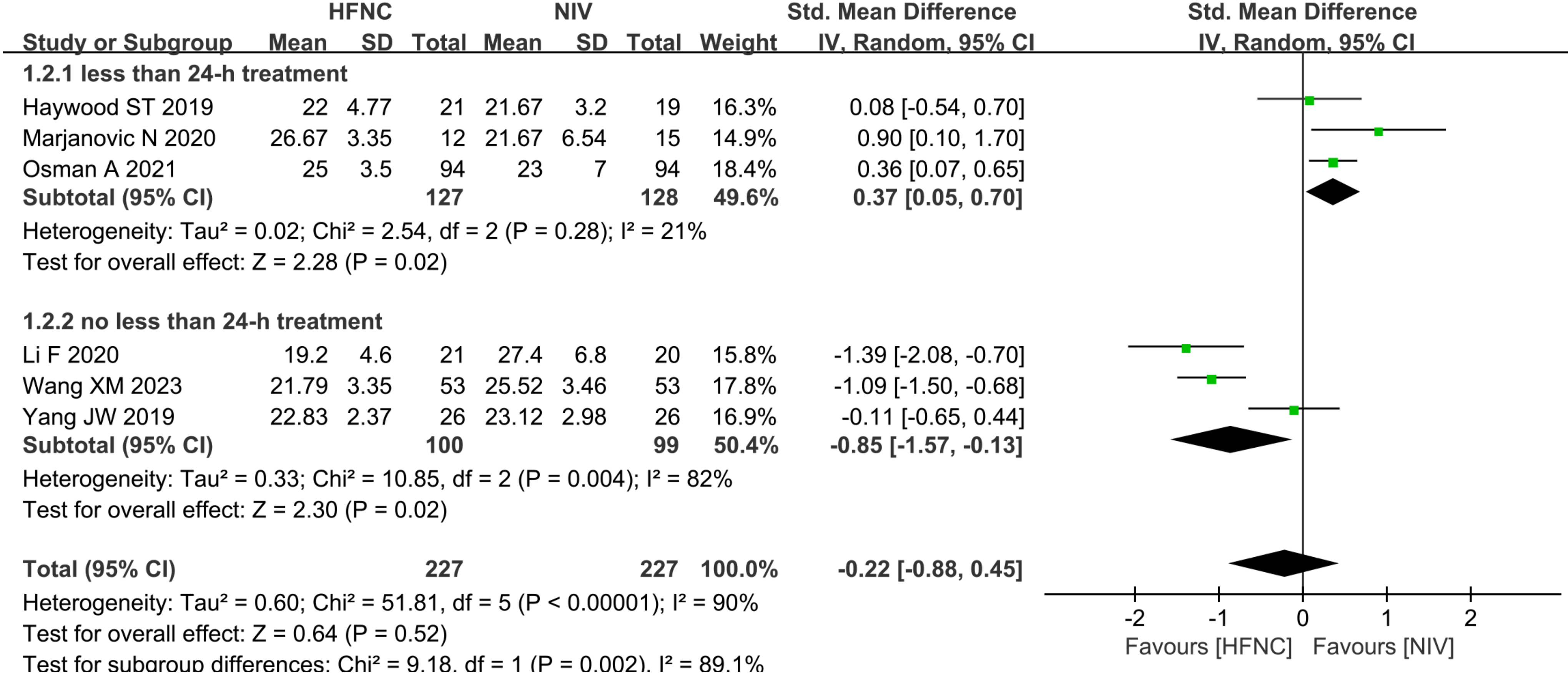

Forest plot of RR-subgroup analysis by risk of bias, P-interaction=0.002, I2=89.1%, HFNC :high-flow nasal cannula oxygen; NIV: noninvasive ventilation; CI: confidence interval; IV: Inverse variance

Supplementary Figure 8-Subgroup stratified analysis of PaO_2

	HFNC			NIV				Std. Mean Difference
Study or Subgroup	Mean	SD	Total	Mean	SD	Total	Weight	IV, Random, 95% CI
**1.3.1 less than 24-h treatment**								
Haywood ST 2019	103	56.43	15	79.25	22.96	16	14.2%	0.54 [-0.18, 1.26]
Marjanovic N 2020	87.67	20.13	12	93.67	44.17	15	13.6%	-0.16 [-0.92, 0.60]
Osman A 2021	163.1	63.31	94	176.52	62.400448	94	20.7%	-0.21 [-0.50, 0.07]
**Subtotal (95% CI)**			**121**			**125**	**48.5%**	**-0.02 [-0.46, 0.42]**
Heterogeneity: Tau² = 0.07; Chi² = 3.68, df = 2 (P = 0.16); I² = 46%								
Test for overall effect: Z = 0.08 (P = 0.93)								
**1.3.2 no less than 24-h treatment**								
Li F 2020	100.3	20	21	94.4	12.9	20	15.7%	0.34 [-0.28, 0.96]
Wang XM 2023	88.42	5.78	53	83.54	5.65	53	19.2%	0.85 [0.45, 1.25]
Yang JW 2019	88.71	8.32	26	83.62	7.16	26	16.6%	0.65 [0.09, 1.20]
**Subtotal (95% CI)**			**100**			**99**	**51.5%**	**0.69 [0.40, 0.97]**
Heterogeneity: Tau² = 0.00; Chi² = 1.85, df = 2 (P = 0.40); I² = 0%								
Test for overall effect: Z = 4.68 (P < 0.00001)								
**Total (95% CI)**			**221**			**224**	**100.0%**	**0.33 [-0.11, 0.78]**
Heterogeneity: Tau² = 0.22; Chi² = 22.42, df = 5 (P = 0.0004); I² = 78%								
Test for overall effect: Z = 1.49 (P = 0.14)								
Test for subgroup differences: Chi² = 6.93, df = 1 (P = 0.008), I² = 85.6%								

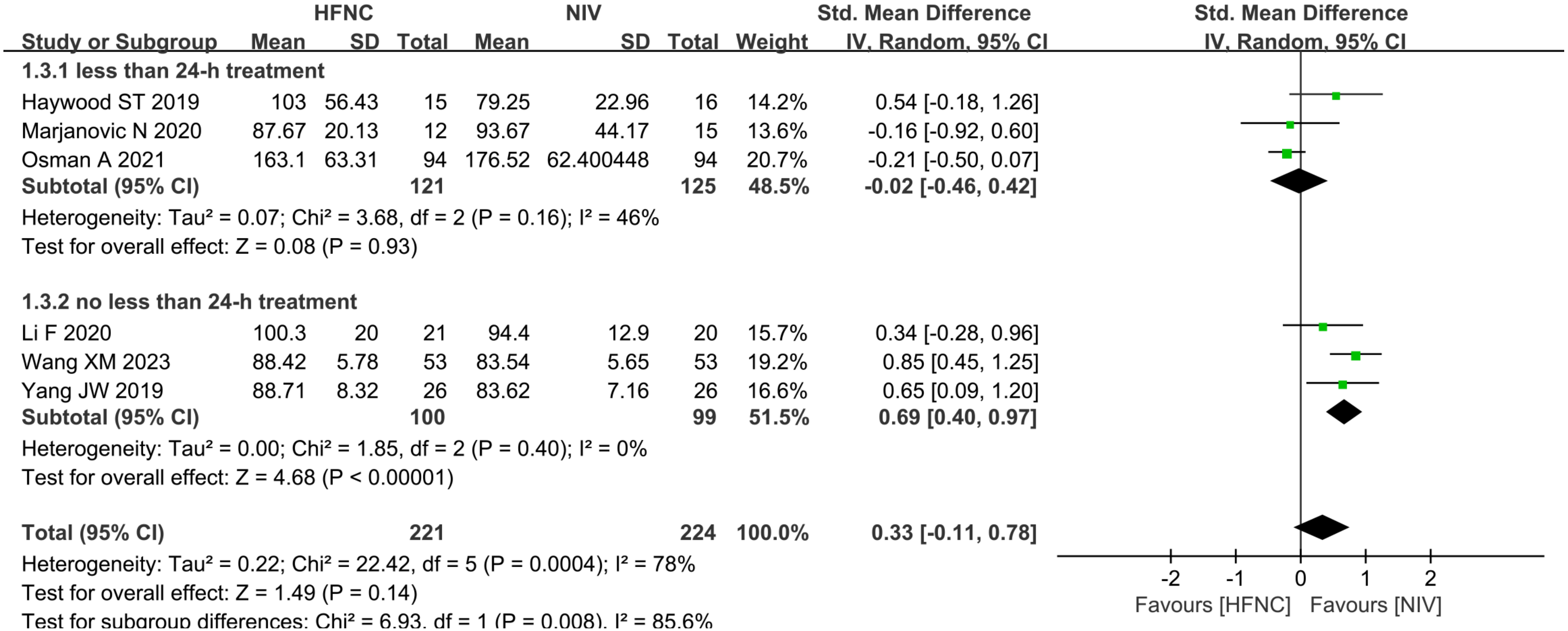

Forest plot of PaO_2-subgroup analysis by risk of bias, P-interaction=0.008, I2=85.6%, HFNC :high-flow nasal cannula oxygen; NIV: noninvasive ventilation; CI:confidence interval; IV: Inverse variance

Supplementary Figure 9-Subgroup stratified analysis of HR

Study or Subgroup	HFNC Mean	HFNC SD	HFNC Total	NIV Mean	NIV SD	NIV Total	Weight	Std. Mean Difference IV, Fixed, 95% CI
**1.5.1 less than 24-h treatment**								
Haywood ST 2019	86.17	16.3	21	82.33	16.82	19	11.5%	0.23 [-0.40, 0.85]
Marjanovic N 2020	94	46.12	12	77.33	24.54	15	7.5%	0.45 [-0.32, 1.22]
Osman A 2021	95	17.49	94	92	20.99	94	54.5%	0.15 [-0.13, 0.44]
**Subtotal (95% CI)**			**127**			**128**	**73.6%**	**0.20 [-0.05, 0.44]**
Heterogeneity: Chi² = 0.52, df = 2 (P = 0.77); I² = 0%								
Test for overall effect: Z = 1.56 (P = 0.12)								
**1.5.2 no less than 24-h treatment**								
Li F 2020	88.4	8.4	21	88.4	12.7	20	11.9%	0.00 [-0.61, 0.61]
Yang JW 2019	78.08	5.09	26	80.75	4.12	26	14.5%	-0.57 [-1.12, -0.01]
**Subtotal (95% CI)**			**47**			**46**	**26.4%**	**-0.31 [-0.72, 0.10]**
Heterogeneity: Chi² = 1.81, df = 1 (P = 0.18); I² = 45%								
Test for overall effect: Z = 1.48 (P = 0.14)								
**Total (95% CI)**			**174**			**174**	**100.0%**	**0.06 [-0.15, 0.27]**
Heterogeneity: Chi² = 6.65, df = 4 (P = 0.16); I² = 40%								
Test for overall effect: Z = 0.58 (P = 0.56)								
Test for subgroup differences: Chi² = 4.31, df = 1 (P = 0.04), I² = 76.8%								

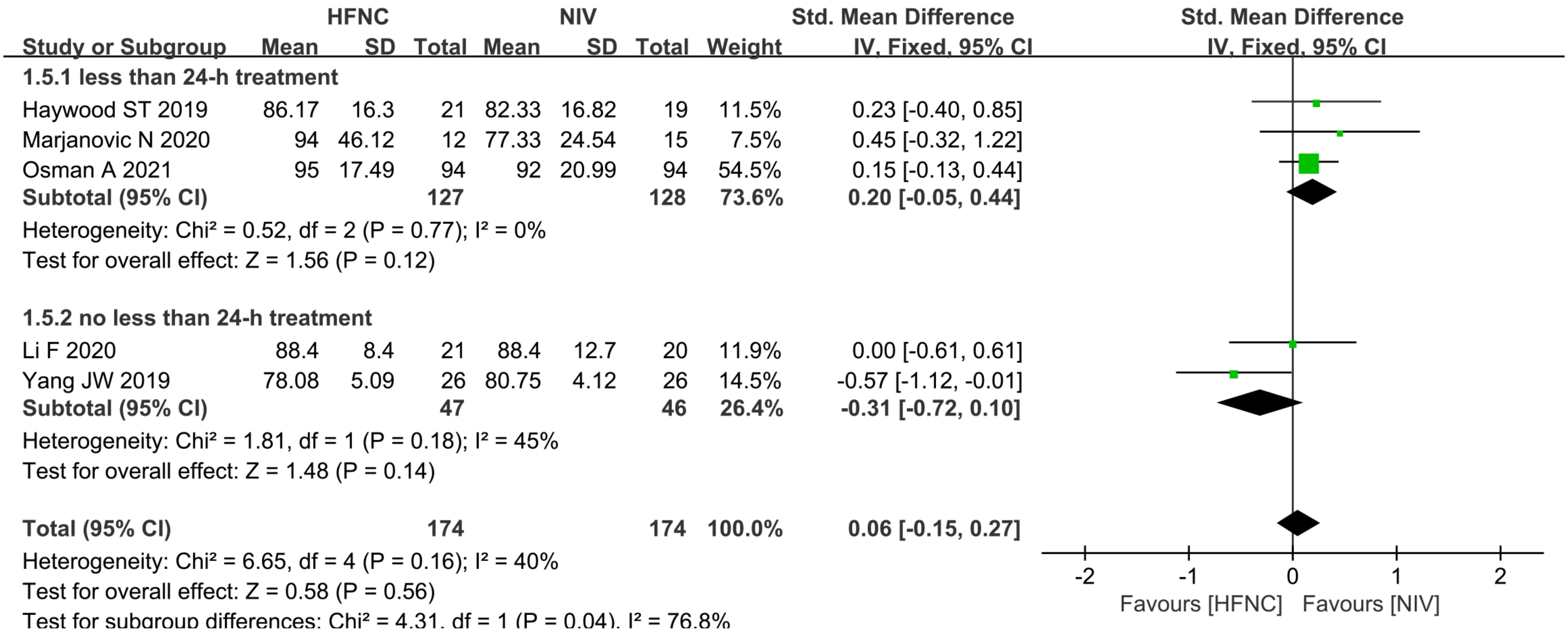

Forest plot of HR-subgroup analysis by risk of bias, P-interaction=0.04, I2=76.8%, HFNC :high-flow nasal cannula oxygen; NIV: noninvasive ventilation; CI:confidence interval; IV: Inverse variance

Supplementary Figure 10-Subgroup stratified analysis of the total length of stay

Study or Subgroup	HFNC Mean	HFNC SD	HFNC Total	COT Mean	COT SD	COT Total	Weight	Std. Mean Difference IV, Random, 95% CI
**1.9.1 1-hour treatment**								
Makdee O 2017	1.1	6.875	63	1.2	4.325	65	25.6%	-0.02 [-0.36, 0.33]
**Subtotal (95% CI)**			**63**			**65**	**25.6%**	**-0.02 [-0.36, 0.33]**
Heterogeneity: Not applicable								
Test for overall effect: Z = 0.10 (P = 0.92)								
**1.9.2 24-hour treatment**								
Li QH 2021	6.32	2.86	39	9.81	3.12	39	24.6%	-1.15 [-1.64, -0.67]
Li YF 2022	10.42	2.01	38	12.49	3.22	38	24.7%	-0.76 [-1.23, -0.30]
Yang FJ 2021	10.23	2.02	64	15.23	3.26	64	25.1%	-1.83 [-2.25, -1.42]
**Subtotal (95% CI)**			**141**			**141**	**74.4%**	**-1.26 [-1.89, -0.62]**
Heterogeneity: Tau² = 0.26; Chi² = 11.77, df = 2 (P = 0.003); I² = 83%								
Test for overall effect: Z = 3.88 (P = 0.0001)								
**Total (95% CI)**			**204**			**206**	**100.0%**	**-0.94 [-1.76, -0.12]**
Heterogeneity: Tau² = 0.65; Chi² = 45.40, df = 3 (P < 0.00001); I² = 93%								
Test for overall effect: Z = 2.24 (P = 0.03)								
Test for subgroup differences: Chi² = 11.29, df = 1 (P = 0.0008), I² = 91.1%								

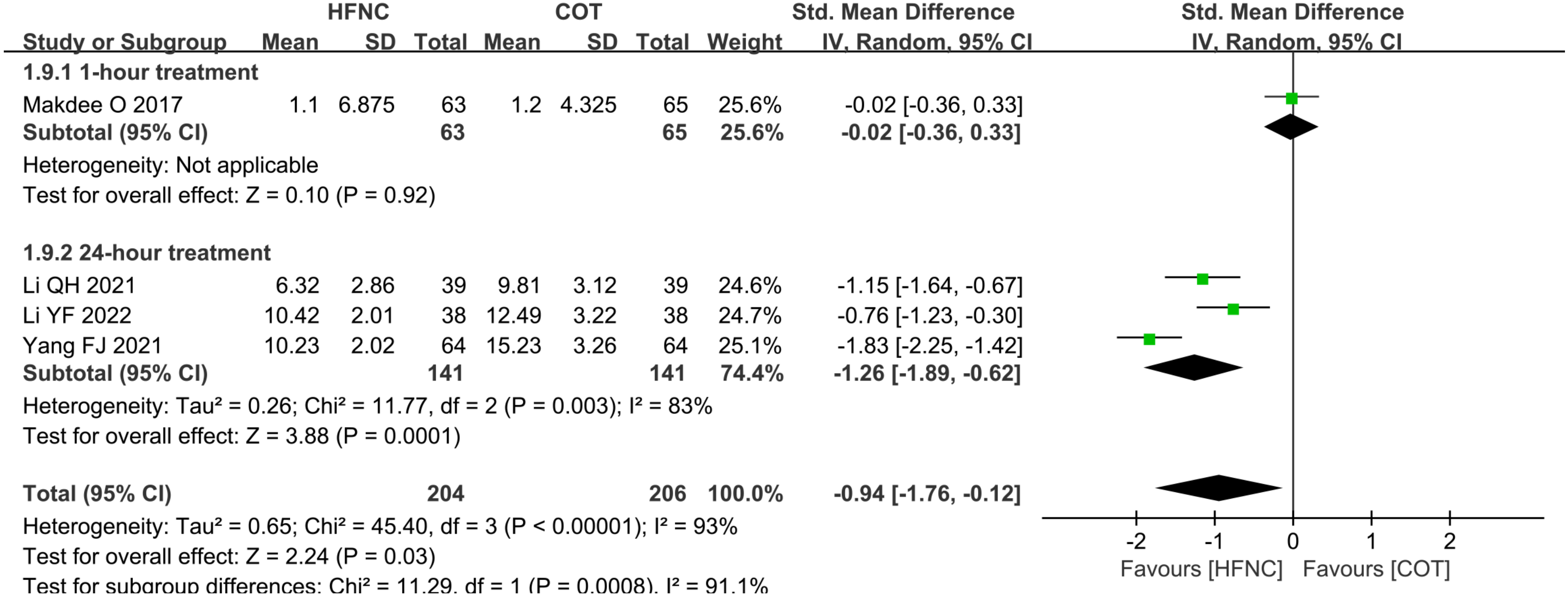

Forest plot of the total length of stay-subgroup analysis by risk of bias, P-interaction=0.0008, I2=91.1%, HFNC :high-flow nasal cannula oxygen; COT: conventional oxygen therapy; CI:confidence interval; IV: Inverse variance

Supplementary Figure 11-Subgroup stratified analysis of pH

Study or Subgroup	HFNC Mean	HFNC SD	HFNC Total	NIV Mean	NIV SD	NIV Total	Weight	Std. Mean Difference IV, Random, 95% CI
**1.6.1 CPAP**								
Osman A 2021	7.39	0.01	94	7.37	0.01	94	26.2%	1.99 [1.64, 2.34]
**Subtotal (95% CI)**			**94**			**94**	**26.2%**	**1.99 [1.64, 2.34]**
Heterogeneity: Not applicable								
Test for overall effect: Z = 11.13 (P < 0.00001)								
**1.6.2 BiPAP**								
Marjanovic N 2020	7.39	0.05	12	7.35	0.08	15	23.9%	0.57 [-0.21, 1.34]
**Subtotal (95% CI)**			**12**			**15**	**23.9%**	**0.57 [-0.21, 1.34]**
Heterogeneity: Not applicable								
Test for overall effect: Z = 1.43 (P = 0.15)								
**1.6.3 Others**								
Haywood ST 2019	7.38	0.06	21	7.38	0.04	19	24.9%	0.00 [-0.62, 0.62]
Li F 2020	7.4	0.1	21	7.4	0.1	20	25.0%	0.00 [-0.61, 0.61]
**Subtotal (95% CI)**			**42**			**39**	**49.9%**	**0.00 [-0.44, 0.44]**
Heterogeneity: Tau² = 0.00; Chi² = 0.00, df = 1 (P = 1.00); I² = 0%								
Test for overall effect: Z = 0.00 (P = 1.00)								
**Total (95% CI)**			**148**			**148**	**100.0%**	**0.66 [-0.50, 1.82]**
Heterogeneity: Tau² = 1.31; Chi² = 51.01, df = 3 (P < 0.00001); I² = 94%								
Test for overall effect: Z = 1.11 (P = 0.27)								
Test for subgroup differences: Chi² = 51.01, df = 2 (P < 0.00001), I² = 96.1%								

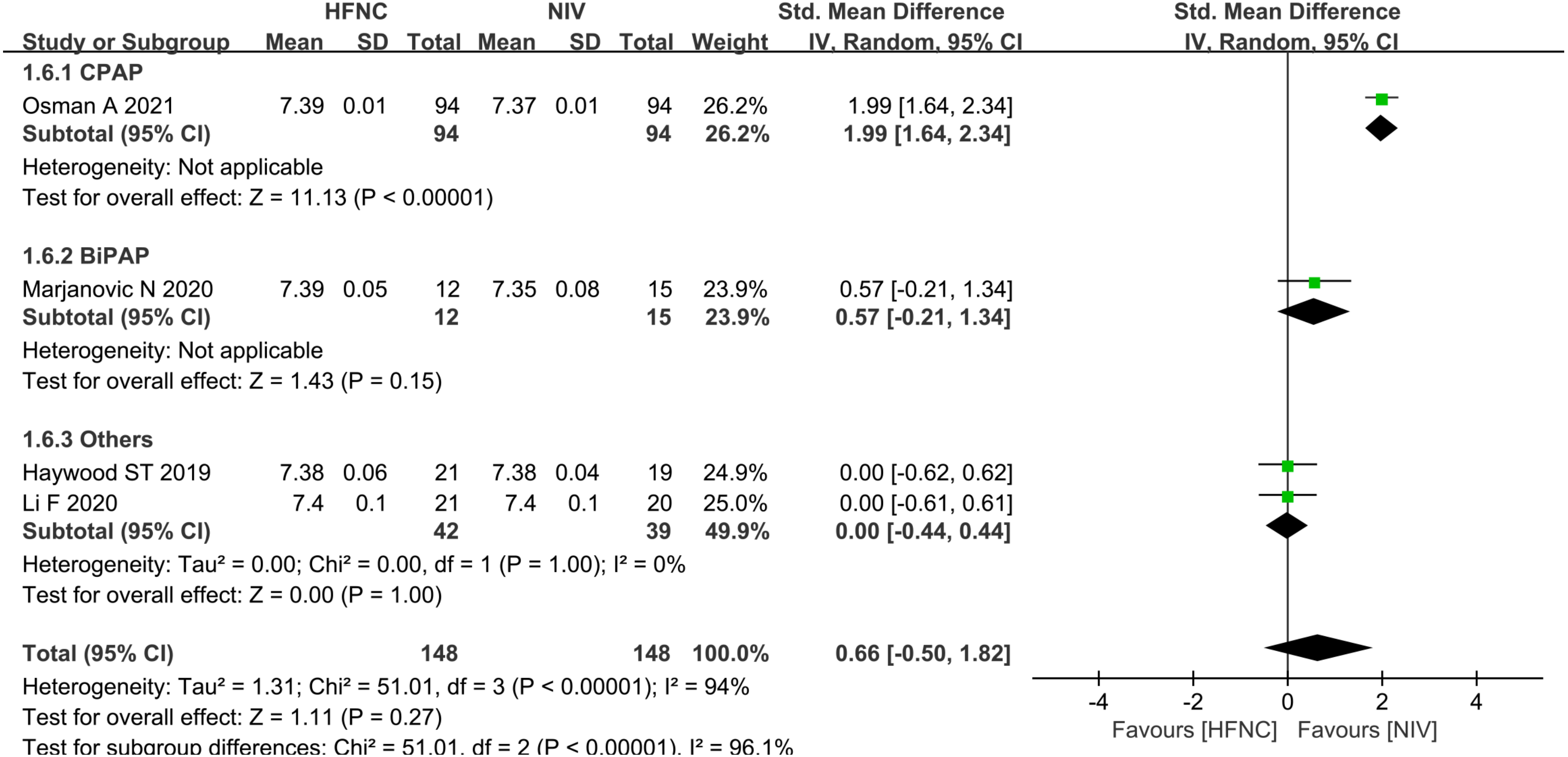

Forest plot of pH-subgroup analysis by risk of bias, P-interaction<0.00001, I2=96.1%, HFNC :high-flow nasal cannulaoxygen; NIV: noninvasive ventilation; CI:confidence interval; IV: Inverse variance

Supplementary Figure 12-Subgroup stratified analysis of dyspnea scores

Study or Subgroup	HFNC Mean	HFNC SD	HFNC Total	NIV Mean	NIV SD	NIV Total	Weight	Std. Mean Difference IV, Random, 95% CI
**1.7.1 CPAP**								
Osman A 2021	2.33	3.76	94	3.33	4.52	94	25.9%	-0.24 [-0.53, 0.05]
Wang XM 2023	2.87	0.36	53	3.66	0.35	53	25.2%	-2.21 [-2.70, -1.72]
**Subtotal (95% CI)**			**147**			**147**	**51.2%**	**-1.21 [-3.14, 0.72]**
Heterogeneity: Tau² = 1.90; Chi² = 46.69, df = 1 (P < 0.00001); I² = 98%								
Test for overall effect: Z = 1.23 (P = 0.22)								
**1.7.2 BiPAP**								
Yang JW 2019	3.02	0.87	26	4.88	0.72	26	24.2%	-2.29 [-3.01, -1.58]
**Subtotal (95% CI)**			**26**			**26**	**24.2%**	**-2.29 [-3.01, -1.58]**
Heterogeneity: Not applicable								
Test for overall effect: Z = 6.32 (P < 0.00001)								
**1.7.3 Others**								
Li F 2020	2.5	0.9	21	2.2	0.9	20	24.7%	0.33 [-0.29, 0.94]
**Subtotal (95% CI)**			**21**			**20**	**24.7%**	**0.33 [-0.29, 0.94]**
Heterogeneity: Not applicable								
Test for overall effect: Z = 1.04 (P = 0.30)								
**Total (95% CI)**			**194**			**193**	**100.0%**	**-1.09 [-2.33, 0.15]**
Heterogeneity: Tau² = 1.52; Chi² = 76.50, df = 3 (P < 0.00001); I² = 96%								
Test for overall effect: Z = 1.73 (P = 0.08)								
Test for subgroup differences: Chi² = 29.94, df = 2 (P < 0.00001), I² = 93.3%								

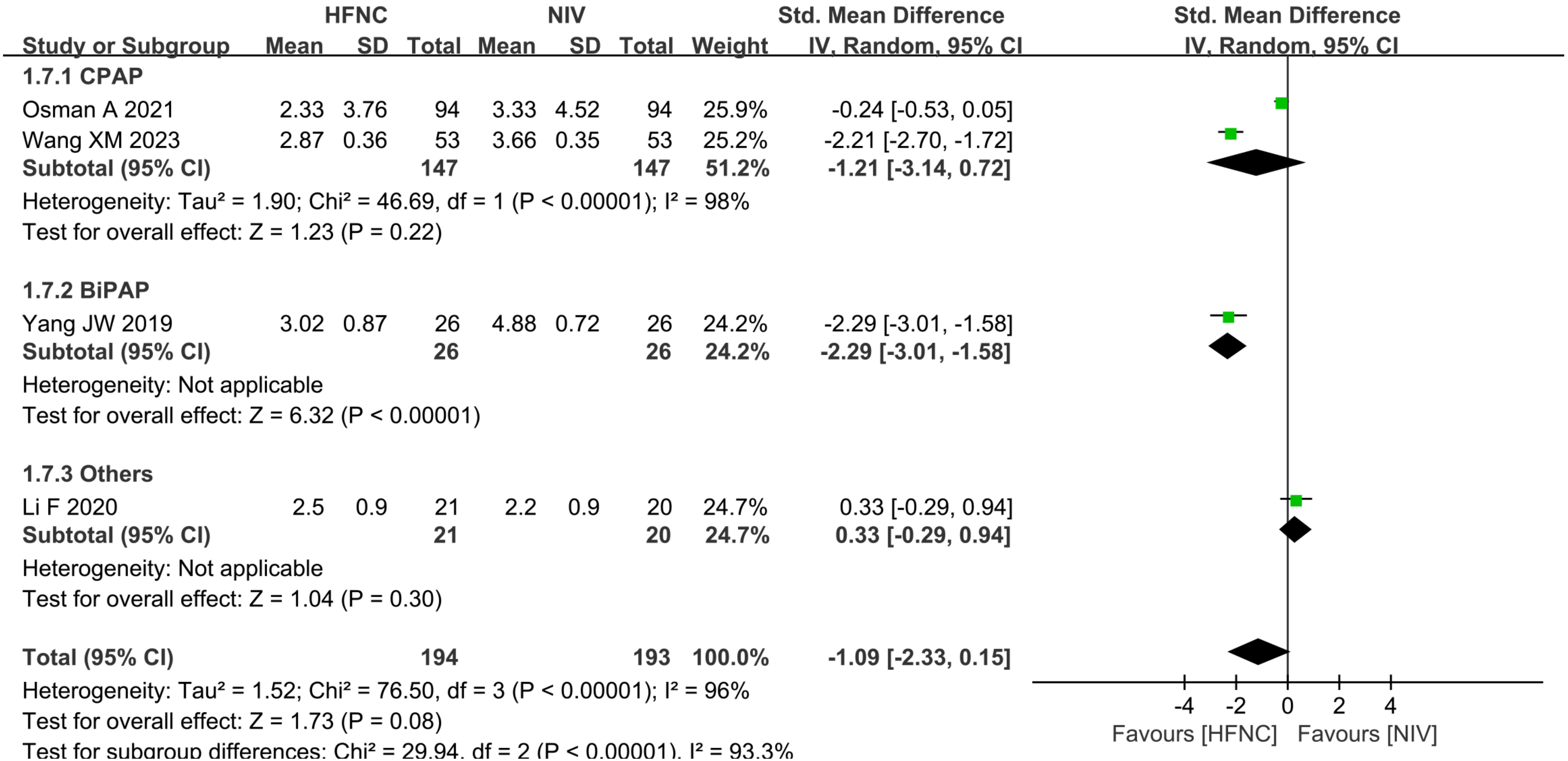

Forest plot of dyspnea scores-subgroup analysis by risk of bias, P-interaction<0.00001, I2=93.3%, HFNC :high-flow nasal cannulaoxygen; NIV: noninvasive ventilation; CI: confidence interval; IV: Inverse variance

Supplementary Figure 13-Subgroup stratified analysis of RR

Study or Subgroup	HFNC Mean	HFNC SD	HFNC Total	NIV Mean	NIV SD	NIV Total	Weight	Std. Mean Difference IV, Random, 95% CI
**1.2.1 Face mask**								
Haywood ST 2019	22	4.77	21	21.67	3.2	19	16.3%	0.08 [-0.54, 0.70]
Marjanovic N 2020	26.67	3.35	12	21.67	6.54	15	14.9%	0.90 [0.10, 1.70]
**Subtotal (95% CI)**			**33**			**34**	**31.2%**	**0.45 [-0.35, 1.25]**
Heterogeneity: Tau² = 0.20; Chi² = 2.53, df = 1 (P = 0.11); I² = 60%								
Test for overall effect: Z = 1.10 (P = 0.27)								
**1.2.2 Helmet**								
Osman A 2021	25	3.5	94	23	7	94	18.4%	0.36 [0.07, 0.65]
**Subtotal (95% CI)**			**94**			**94**	**18.4%**	**0.36 [0.07, 0.65]**
Heterogeneity: Not applicable								
Test for overall effect: Z = 2.45 (P = 0.01)								
**1.2.3 Others**								
Li F 2020	19.2	4.6	21	27.4	6.8	20	15.8%	-1.39 [-2.08, -0.70]
Wang XM 2023	21.79	3.35	53	25.52	3.46	53	17.8%	-1.09 [-1.50, -0.68]
Yang JW 2019	22.83	2.37	26	23.12	2.98	26	16.9%	-0.11 [-0.65, 0.44]
**Subtotal (95% CI)**			**100**			**99**	**50.4%**	**-0.85 [-1.57, -0.13]**
Heterogeneity: Tau² = 0.33; Chi² = 10.85, df = 2 (P = 0.004); I² = 82%								
Test for overall effect: Z = 2.30 (P = 0.02)								
**Total (95% CI)**			**227**			**227**	**100.0%**	**-0.22 [-0.88, 0.45]**
Heterogeneity: Tau² = 0.60; Chi² = 51.81, df = 5 (P < 0.00001); I² = 90%								
Test for overall effect: Z = 0.64 (P = 0.52)								
Test for subgroup differences: Chi² = 9.61, df = 2 (P = 0.008), I² = 79.2%								

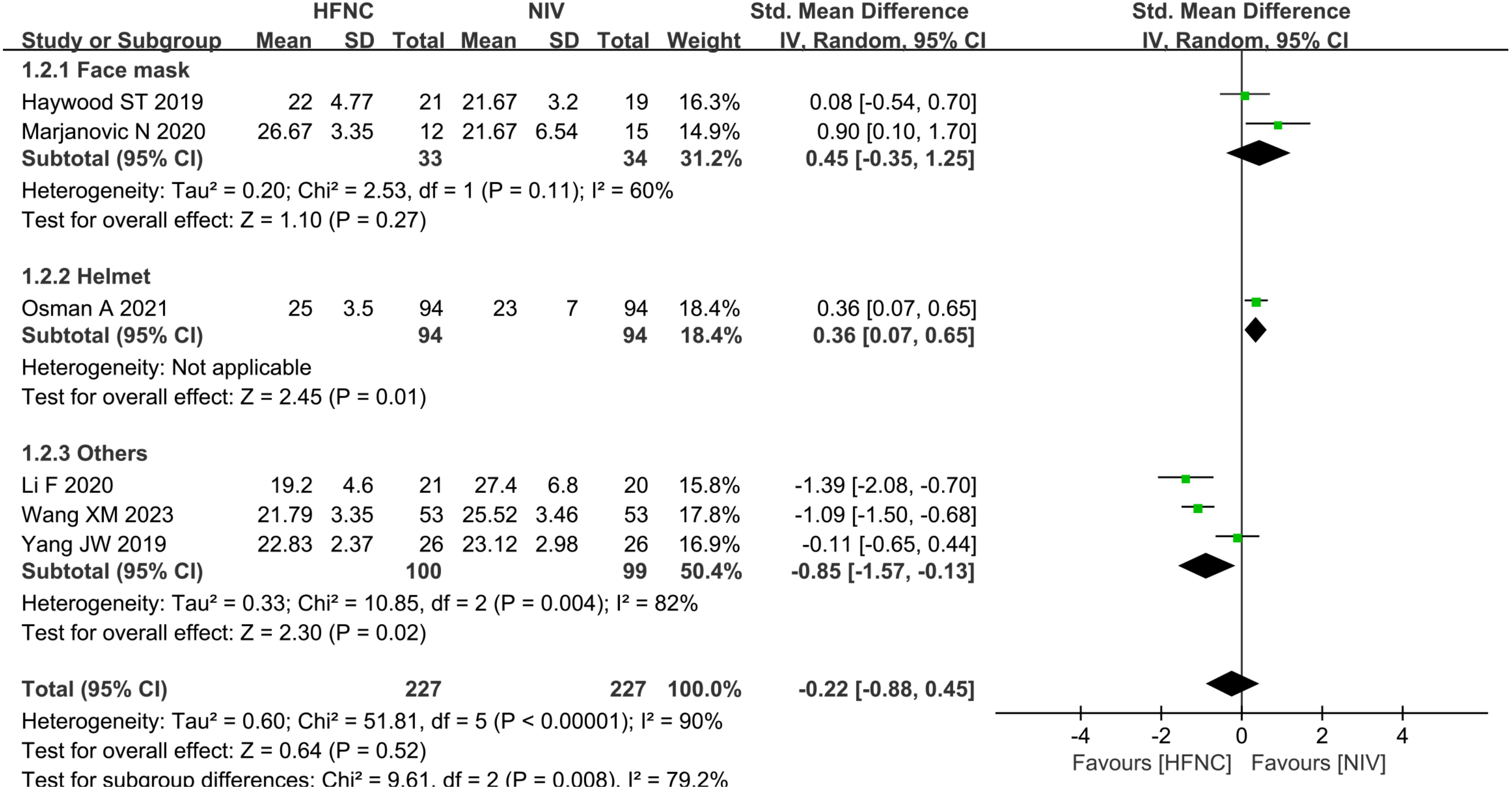

Forest plot of RR-subgroup analysis by risk of bias, P-interaction=0.008, I2=79.2%, HFNC :high-flow nasal cannulaoxygen; NIV: noninvasive ventilation; CI:confidence interval; IV: Inverse variance

Supplementary Figure 14-Subgroup stratified analysis of pH

Study or Subgroup	HFNC Mean	HFNC SD	HFNC Total	NIV Mean	NIV SD	NIV Total	Weight	Std. Mean Difference IV, Random, 95% CI
**1.6.1 Face mask**								
Haywood ST 2019	7.38	0.06	21	7.38	0.04	19	24.9%	0.00 [-0.62, 0.62]
Marjanovic N 2020	7.39	0.05	12	7.35	0.08	15	23.9%	0.57 [-0.21, 1.34]
**Subtotal (95% CI)**			**33**			**34**	**48.9%**	**0.23 [-0.31, 0.78]**
Heterogeneity: $Tau^2 = 0.03$; $Chi^2 = 1.25$, df = 1 (P = 0.26); $I^2 = 20\%$								
Test for overall effect: Z = 0.84 (P = 0.40)								
**1.6.2 Helmet**								
Osman A 2021	7.39	0.01	94	7.37	0.01	94	26.2%	1.99 [1.64, 2.34]
**Subtotal (95% CI)**			**94**			**94**	**26.2%**	**1.99 [1.64, 2.34]**
Heterogeneity: Not applicable								
Test for overall effect: Z = 11.13 (P < 0.00001)								
**1.6.3 Others**								
Li F 2020	7.4	0.1	21	7.4	0.1	20	25.0%	0.00 [-0.61, 0.61]
**Subtotal (95% CI)**			**21**			**20**	**25.0%**	**0.00 [-0.61, 0.61]**
Heterogeneity: Not applicable								
Test for overall effect: Z = 0.00 (P = 1.00)								
**Total (95% CI)**			**148**			**148**	**100.0%**	**0.66 [-0.50, 1.82]**
Heterogeneity: $Tau^2 = 1.31$; $Chi^2 = 51.01$, df = 3 (P < 0.00001); $I^2 = 94\%$								
Test for overall effect: Z = 1.11 (P = 0.27)								
Test for subgroup differences: $Chi^2 = 46.33$, df = 2 (P < 0.00001), $I^2 = 95.7\%$								

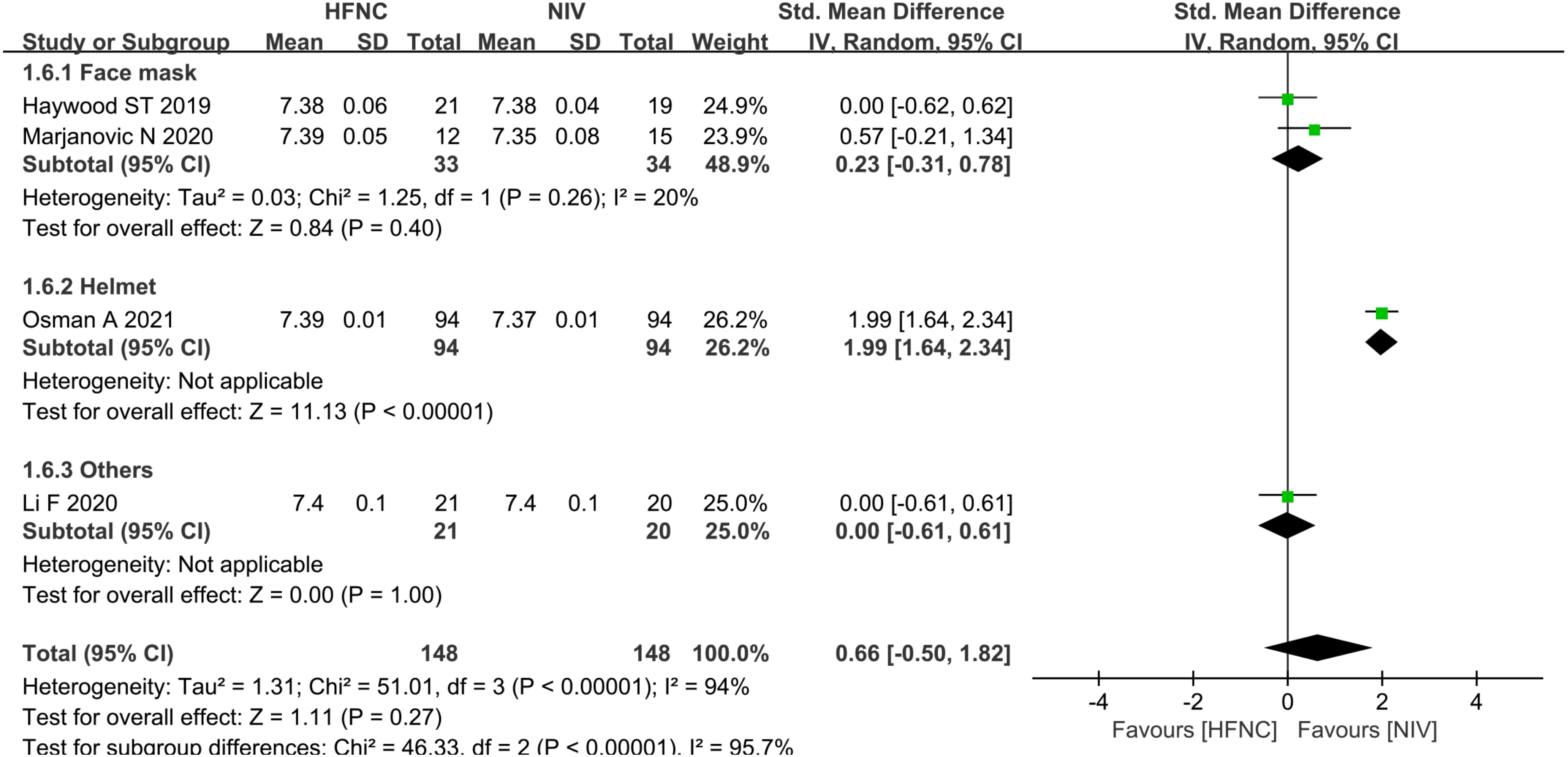

Forest plot of pH-subgroup analysis by risk of bias, P-interaction<0.00001, I2=95.7%, HFNC :high-flow nasal cannulaoxygen; NIV: noninvasive ventilation; CI: confidence interval; IV: Inverse variance

Supplementary Appendix 1. Detailed search strategies and extraction strategy

Detailed search strategies of PubMed, Embase, Web of Science, MEDLINE and the Cochrane Library

PubMed

((((((((((((((("Heart Failure"[Mesh]) OR (Cardiac Failure)) OR (Myocardial Failure)) OR (Heart Failure, Left-Sided)) OR (Heart Failure, Left Sided)) OR (Left-Sided Heart Failure)) OR (Left Sided Heart Failure)) OR (Heart Failure, Right-Sided)) OR (Heart Failure, Right Sided)) OR (Right-Sided Heart Failure)) OR (Right Sided Heart Failure)) OR (Congestive Heart Failure)) OR (Heart Failure, Congestive)) OR (Heart Decompensation)) OR (Decompensation, Heart)) AND (((((((((HFNC) OR (nasal high flow oxygen)) OR (high flow nasal cannula)) OR (high flow nasal therapy)) OR (high flow nasal oxygen)) OR (high flow oxygen therapy)) OR (high flow therapy)) OR (optiflow)) OR (nasal highflow))) AND (((((((((((("Noninvasive Ventilation"[Mesh]) OR (Noninvasive Ventilations)) OR (Ventilation, Noninvasive)) OR (Ventilations, Noninvasive)) OR (Non-Invasive Ventilation)) OR (Non-Invasive Ventilations)) OR (Ventilation, Non-Invasive)) OR (Ventilations, Non-Invasive)) OR (Non Invasive Ventilation)) OR (Non Invasive Ventilations)) OR (Ventilation, Non Invasive)) OR (Ventilations, Non Invasive)) OR ((((((((((("Oxygen Inhalation Therapy"[Mesh]) OR (Inhalation Therapy, Oxygen)) OR (Inhalation Therapies, Oxygen)) OR (Oxygen Inhalation Therapies)) OR (Therapies, Oxygen Inhalation)) OR (Therapy, Oxygen Inhalation)) OR (conventional oxygen therapy)) OR (COT)) OR (oxygen supplementation)) OR (oxygen delivery)) OR (venturi mask)))

304

Embase

#7 #5 AND #6 51

#6 #3 OR #4 32,905

#5 #1 AND #2 184

#4 'oxygen inhalation therapy':ab,ti OR 'inhalation therapy, oxygen':ab,ti OR 'inhalation therapies, oxygen':ab,ti OR 'oxygen inhalation therapies':ab,ti OR 'therapies, oxygen inhalation':ab,ti OR 'therapy, oxygen inhalation':ab,ti OR 'conventional oxygen therapy':ab,ti OR cot:ab,ti OR 'oxygen supplementation':ab,ti OR 'oxygen delivery':ab,ti OR 'venturi mask':ab,ti 19,056

#3 'noninvasive ventilation':ab,ti OR 'noninvasive ventilations':ab,ti OR 'ventilation, noninvasive':ab,ti OR 'ventilations, noninvasive':ab,ti OR 'non-invasive ventilation':ab,ti OR 'non-invasive ventilations':ab,ti OR 'ventilation, non-invasive':ab,ti OR 'ventilations, non-invasive':ab,ti OR 'non invasive ventilation':ab,ti OR 'non invasive ventilations':ab,ti OR 'ventilation, non invasive':ab,ti OR 'ventilations, non invasive':ab,ti 14,268

#2 hfnc:ab,ti OR 'nasal high flow oxygen':ab,ti OR 'high flow nasal cannula':ab,ti OR 'high flow nasal therapy':ab,ti OR 'high flow nasal oxygen':ab,ti OR 'high flow oxygen therapy':ab,ti OR 'high flow therapy':ab,ti OR optiflow:ab,ti OR 'nasal highflow':ab,ti 6110

#1 'heart failure':ab,ti OR 'cardiac failure':ab,ti OR 'myocardial failure':ab,ti OR 'heart failure, left-sided':ab,ti OR 'heart failure, left sided':ab,ti OR 'left-sided heart failure':ab,ti OR 'left sided heart failure':ab,ti OR 'heart failure, right-sided':ab,ti OR 'heart failure, right sided':ab,ti OR 'right-sided heart failure':ab,ti OR 'right sided heart failure':ab,ti OR 'congestive heart failure':ab,ti

OR 'heart failure, congestive':ab,ti OR 'heart decompensation':ab,ti OR 'decompensation, heart':ab,ti 368353

51

Web of Science

#1 TS= (Heart Failure OR Cardiac Failure OR Myocardial Failure OR Heart Failure, Left-Sided OR Heart Failure, Left Sided OR Left-Sided Heart Failure OR Left Sided Heart Failure OR Heart Failure, Right-Sided OR Heart Failure, Right Sided OR Right-Sided Heart Failure OR Right Sided Heart Failure OR Congestive Heart Failure OR Heart Failure, Congestive OR Heart Decompensation OR Decompensation, Heart)

#2 TS= (HFNC OR nasal high flow oxygen OR high flow nasal cannula OR high flow nasal therapy OR high flow nasal oxygen OR high flow oxygen therapy OR high flow therapy OR optiflow OR nasal highflow)

#3 TS= (Noninvasive Ventilation OR Noninvasive Ventilations OR Ventilation, Noninvasive OR Ventilations, Noninvasive OR Non-Invasive Ventilation OR Non-Invasive Ventilations OR Ventilation, Non-Invasive OR Ventilations, Non-Invasive OR Non Invasive Ventilation OR Non Invasive Ventilations OR Ventilation, Non Invasive OR Ventilations, Non Invasive)

#4 TS= (Oxygen Inhalation Therapy OR Inhalation Therapy, Oxygen OR Inhalation Therapies, Oxygen OR Oxygen Inhalation Therapies OR Therapies, Oxygen Inhalation OR Therapy, Oxygen Inhalation OR conventional oxygen therapy OR COT OR oxygen supplementation OR oxygen delivery OR venturi mask)

#1 AND #2 AND (#3 OR #4)

541

MEDLINE

1 exp Heart Failure/
2 exp Noninvasive Ventilation/
3 exp Oxygen Inhalation Therapy/
4 ((high frequency or high flow) adj5 nasal).tw.
5 ((high frequency or high flow) adj5 oxygen).tw.
6 (high-flow adj5 nasal).tw.
7 (nasal adj5 high flow).tw.
8 (hfnc or hfnp or hhfnox or hfno).tw.
9 4 or 5 or 6 or 7 or 8
10 1 and 9
11 2 or 3

12 10 and 11

18

Cochrane Library

#1 MeSH descriptor: [Heart Failure] explode all trees
#2 Heart Failure OR Cardiac Failure OR Myocardial Failure OR Heart Failure, Left-Sided OR Heart Failure, Left Sided OR Left-Sided Heart Failure OR Left Sided Heart Failure OR Heart Failure, Right-Sided OR Heart Failure, Right Sided OR Right-Sided Heart Failure OR Right Sided Heart Failure OR Congestive Heart Failure OR Heart Failure, Congestive OR Heart Decompensation OR Decompensation, Heart
#3 HFNC OR nasal high flow oxygen OR high flow nasal cannula OR high flow nasal therapy OR high flow nasal oxygen OR high flow oxygen therapy OR high flow therapy OR optiflow OR nasal highflow
#4 MeSH descriptor: [Noninvasive Ventilation] explode all trees
#5 Noninvasive Ventilation OR Noninvasive Ventilations OR Ventilation, Noninvasive OR Ventilations, Noninvasive OR Non-Invasive Ventilation OR Non-Invasive Ventilations OR Ventilation, Non-Invasive OR Ventilations, Non-Invasive OR Non Invasive Ventilation OR Non Invasive Ventilations OR Ventilation, Non Invasive OR Ventilations, Non Invasive
#6 MeSH descriptor: [Oxygen Inhalation Therapy] explode all trees
#7 Oxygen Inhalation Therapy OR Inhalation Therapy, Oxygen OR Inhalation Therapies, Oxygen OR Oxygen Inhalation Therapies OR Therapies, Oxygen Inhalation OR Therapy, Oxygen Inhalation OR conventional oxygen therapy OR COT OR oxygen supplementation OR oxygen delivery OR venturi mask
#8 #1 or #2
#9 #3 and #8
#10 #4 or #5 or #6 or #7
#11 #9 and #10

223

CNKI

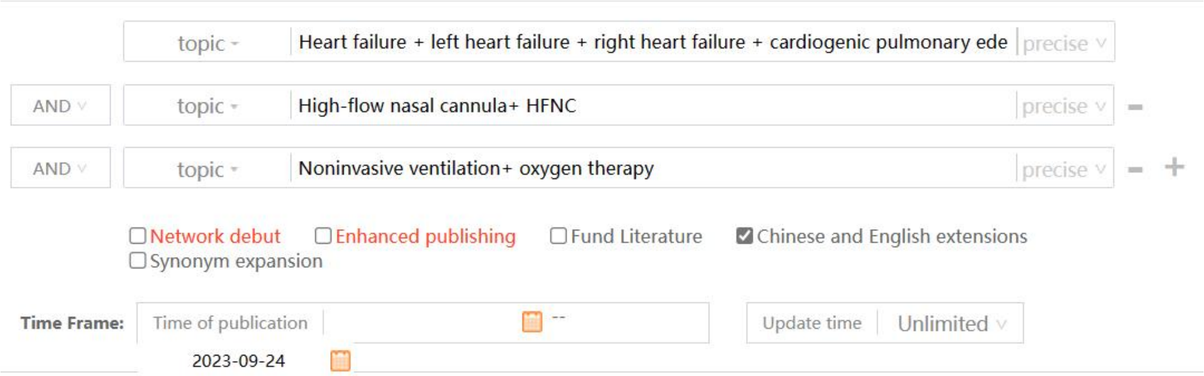

67

Wanfang

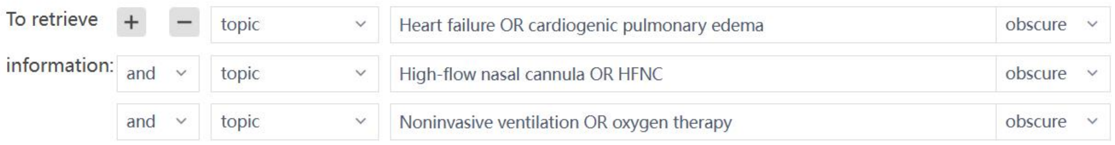

45

VIP

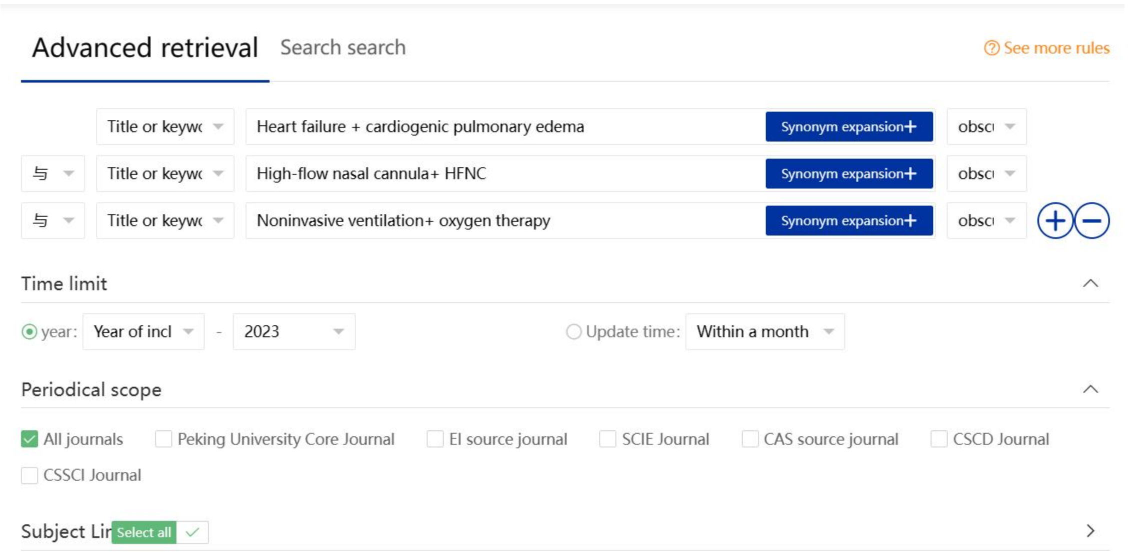

39

Data extraction strategy

Two researchers (Liming Yan and Ye Lu) first screened the trials independently. During title and abstract screening, duplicated and non-randomized controlled studies were deleted. Where the facility was available, we used the "explode" function in those databases. Bibliographies of included papers and relevant reviews were searched for further possible titles and the process was repeated until no new titles were found. Where the same data were presented in more than one publication we used the primary (first) publication. Then, through full-text retrieval, the studies that met the inclusion criteria were obtained. Disagreements could be resolved by discussion with the third researcher (Xiaoming Zhou). A standardized data extraction table was used to extract the literature, including the first author, the publication year, the research country, the study type, the risk of bias assessment of the randomized controlled trial (RCT), sample size of the research object, basic information of the patients (age, sex), intervention and control measures, and the evaluation outcome indicators.

Supplementary Appendix 2. Sensitivity analysis and funnel plot

Sensitivity analysis of intubation between high-flow nasal cannula oxygen (HFNC) and conventional oxygen therapy (COT).

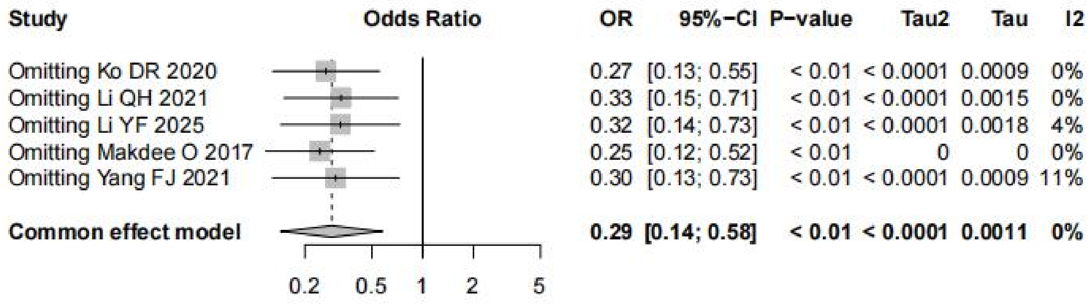

Sensitivity analysis of RR between high-flow nasal cannula oxygen (HFNC) and conventional oxygen therapy (COT).

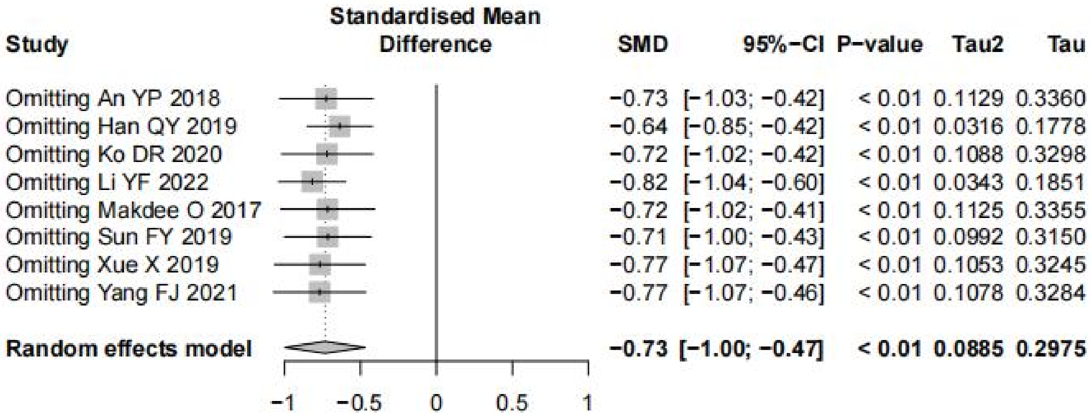

Sensitivity analysis of RR between high-flow nasal cannula oxygen (HFNC) and noninvasive ventilation (NIV)

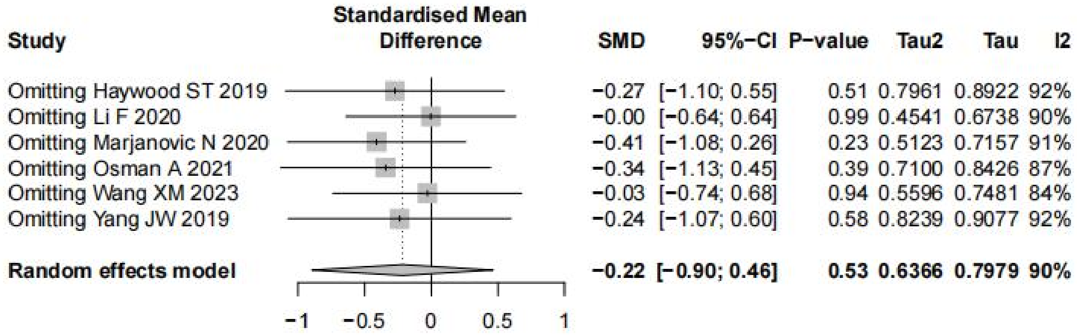

Sensitivity analysis of PaO2 between high-flow nasal cannula oxygen (HFNC) and conventional oxygen therapy (COT).

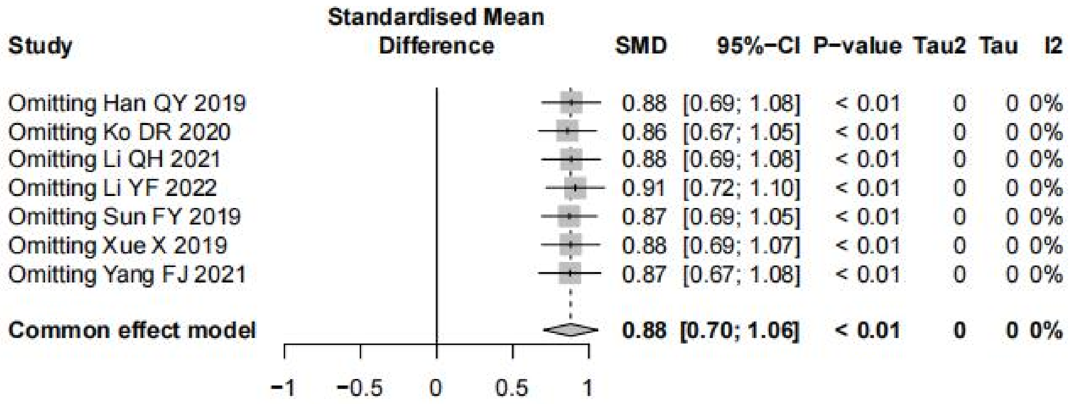

Sensitivity analysis of PaO2 between high-flow nasal cannula oxygen (HFNC) and noninvasive ventilation (NIV).

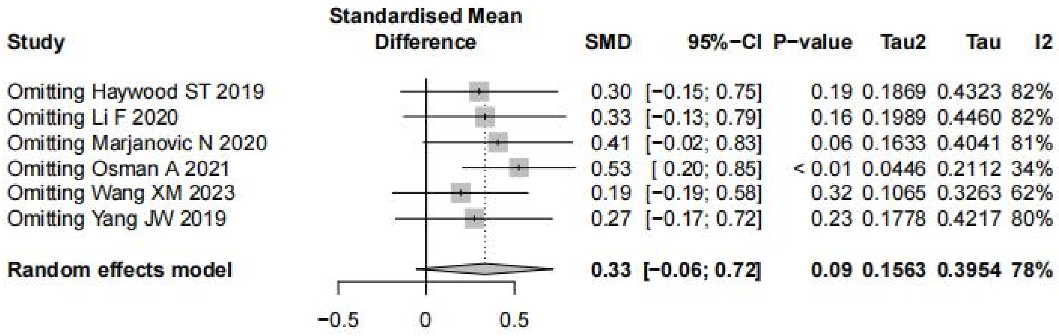

Sensitivity analysis of PaCO2 between high-flow nasal cannula oxygen (HFNC) and conventional oxygen therapy (COT).

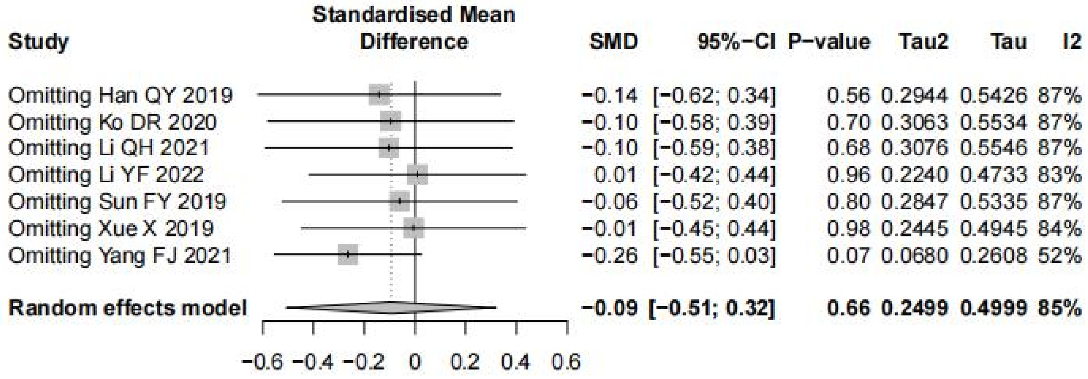

Sensitivity analysis of PaCO2 between high-flow nasal cannula oxygen (HFNC) and noninvasive ventilation (NIV).

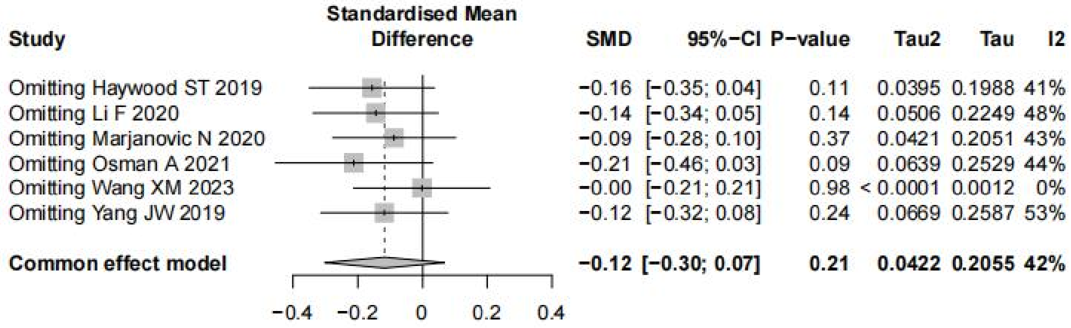

Sensitivity analysis of SpO2 between high-flow nasal cannula oxygen (HFNC) and conventional oxygen therapy (COT).

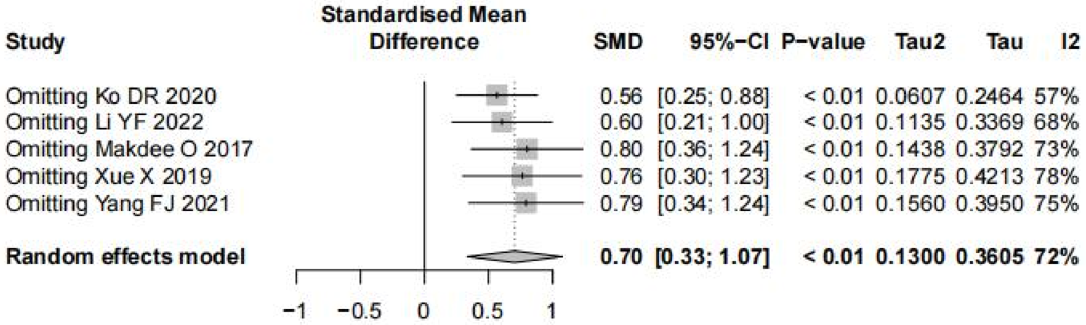

Sensitivity analysis of HR between high-flow nasal cannula oxygen (HFNC) and conventional oxygen therapy (COT).

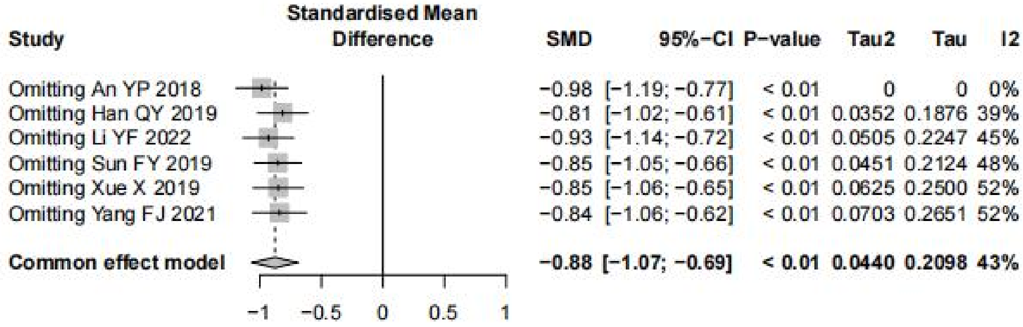

Sensitivity analysis of HR between high-flow nasal cannula oxygen (HFNC) and noninvasive ventilation (NIV)

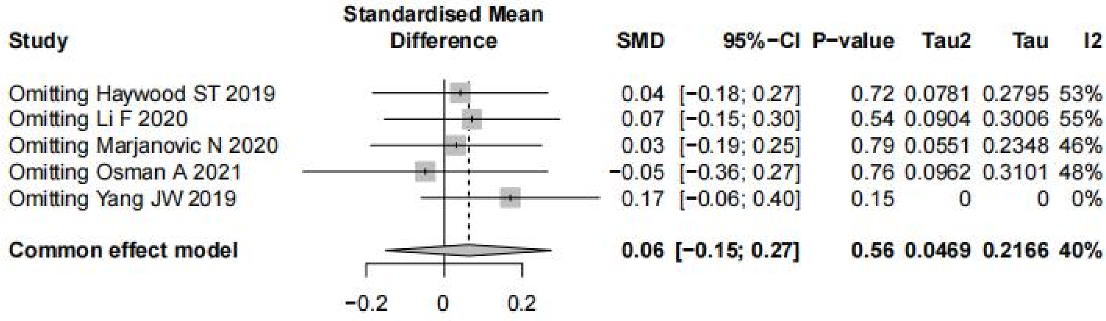

Funnel plot

Funnel plot comparing of intubation between high-flow nasal cannula oxygen (HFNC) and conventional oxygen therapy (COT).

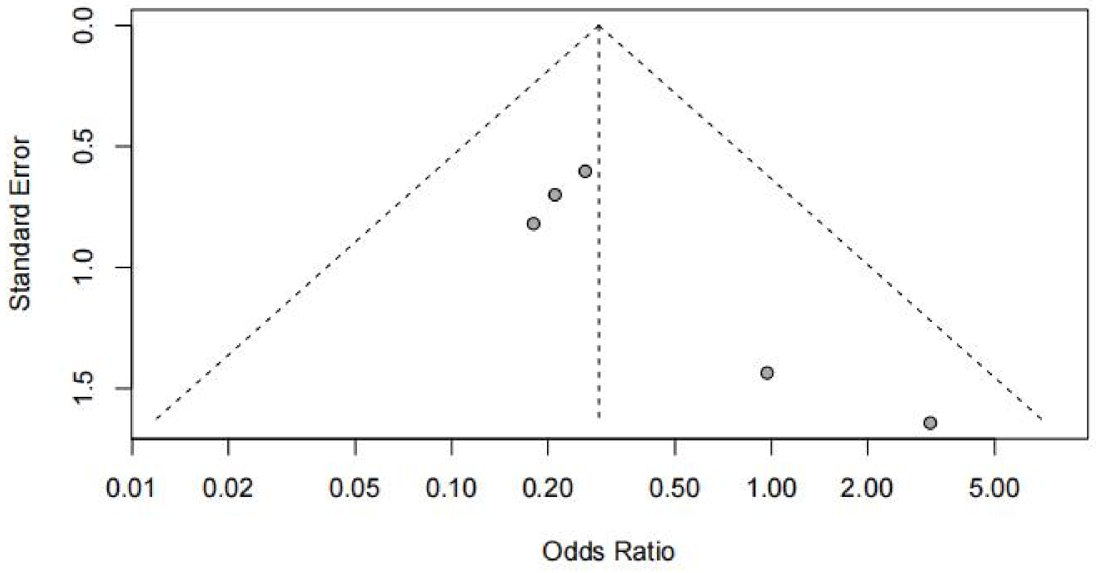

Funnel plot comparing of RR between high-flow nasal cannula oxygen (HFNC) and conventional oxygen therapy (COT).

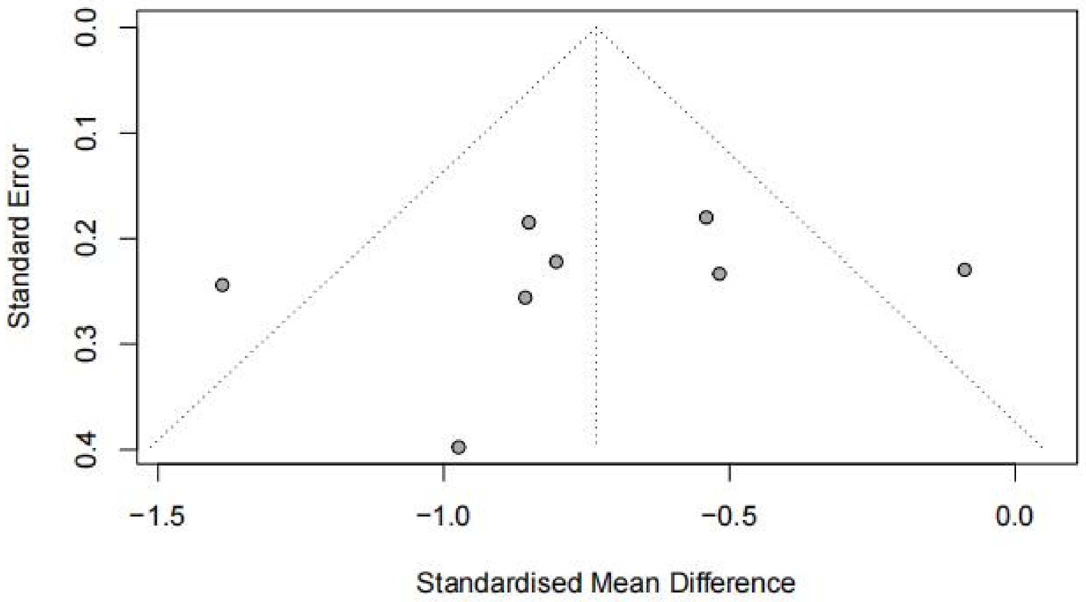

Funnel plot comparing of RR between high-flow nasal cannula oxygen (HFNC) and noninvasive ventilation (NIV).

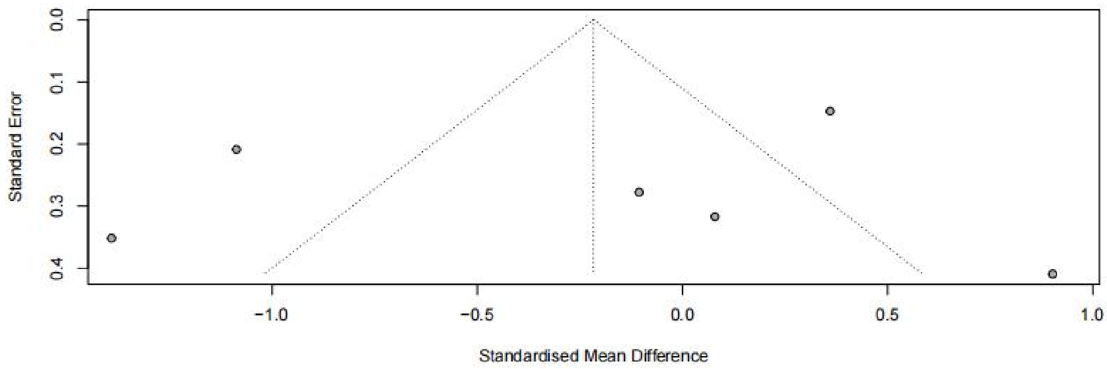

Funnel plot comparing of PaO2 between high-flow nasal cannula oxygen (HFNC) and conventional oxygen therapy (COT).

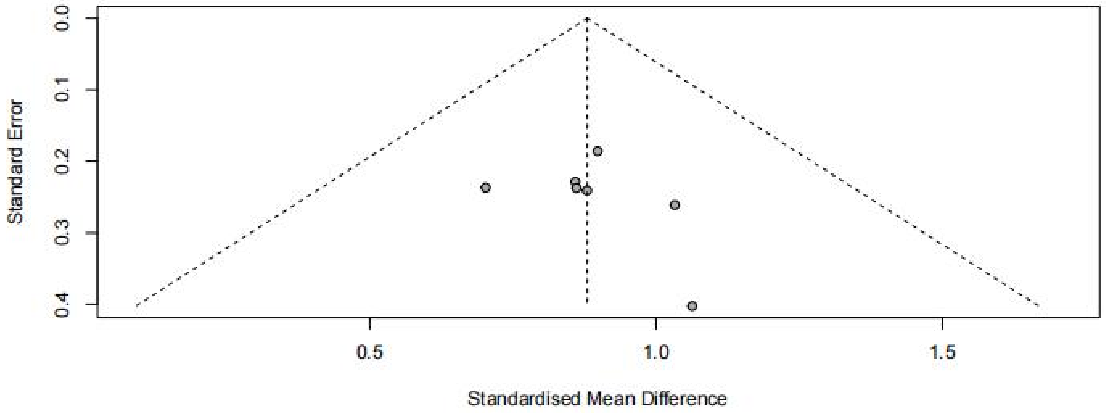

Funnel plot comparing of PaO2 between high-flow nasal cannula oxygen (HFNC) and noninvasive ventilation (NIV).

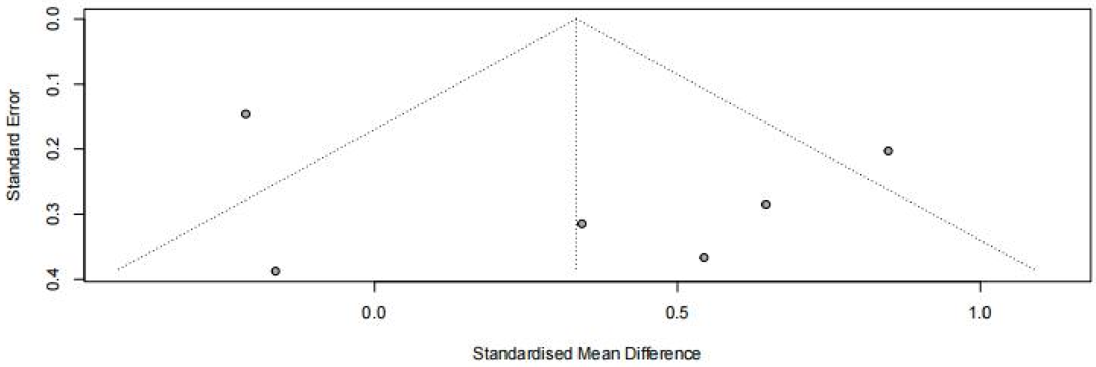

Funnel plot comparing of PaCO2 between high-flow nasal cannula oxygen (HFNC) and conventional oxygen therapy (COT).

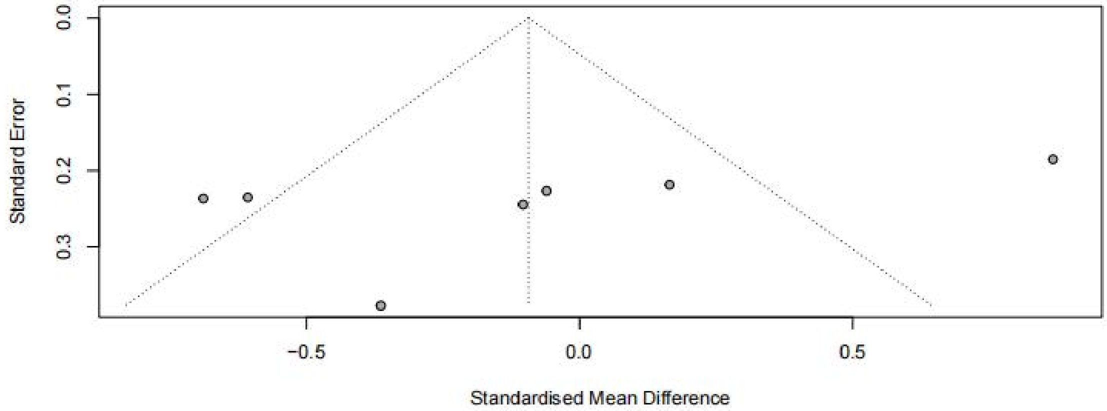

Funnel plot comparing of PaCO2 between high-flow nasal cannula oxygen (HFNC) and noninvasive ventilation (NIV).

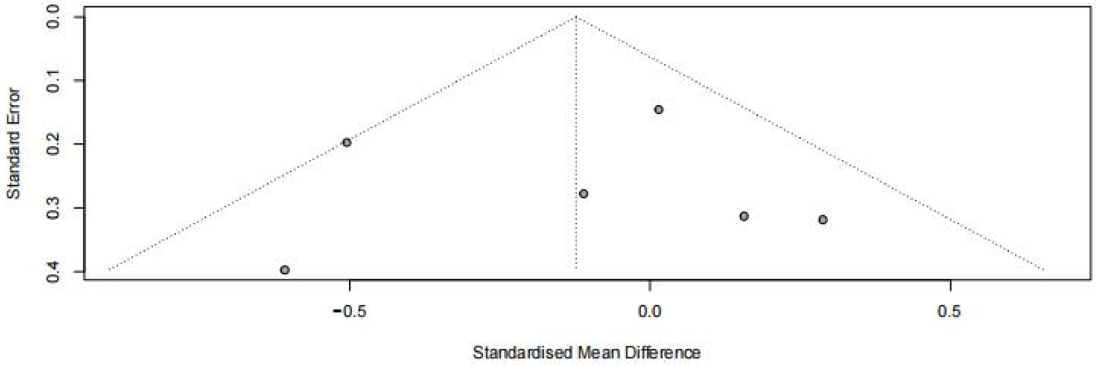

Funnel plot comparing of SpO2 between high-flow nasal cannula oxygen (HFNC) and conventional oxygen therapy (COT).

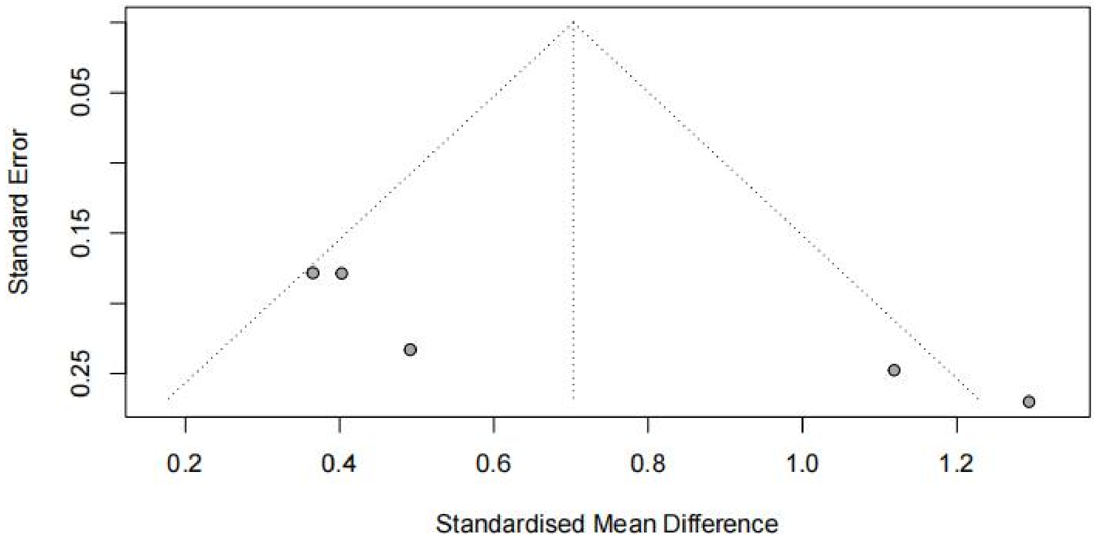

Funnel plot comparing of HR between high-flow nasal cannula oxygen (HFNC) and conventional oxygen therapy (COT).

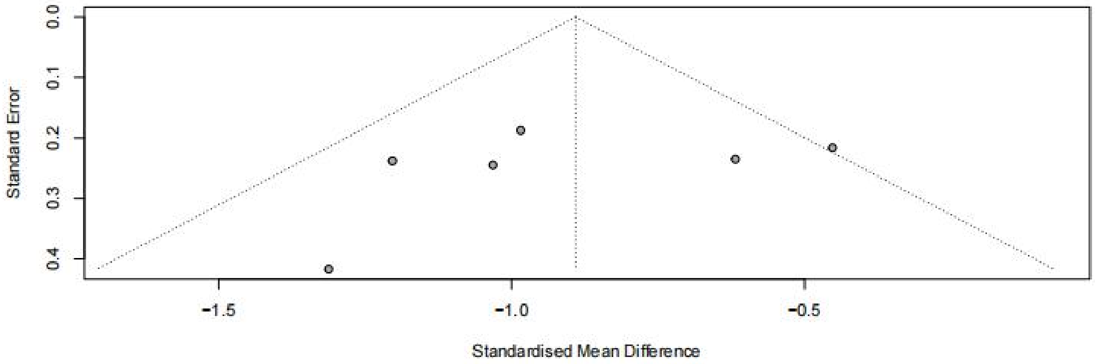

Funnel plot comparing of HR between high-flow nasal cannula oxygen (HFNC) and noninvasive ventilation (NIV).

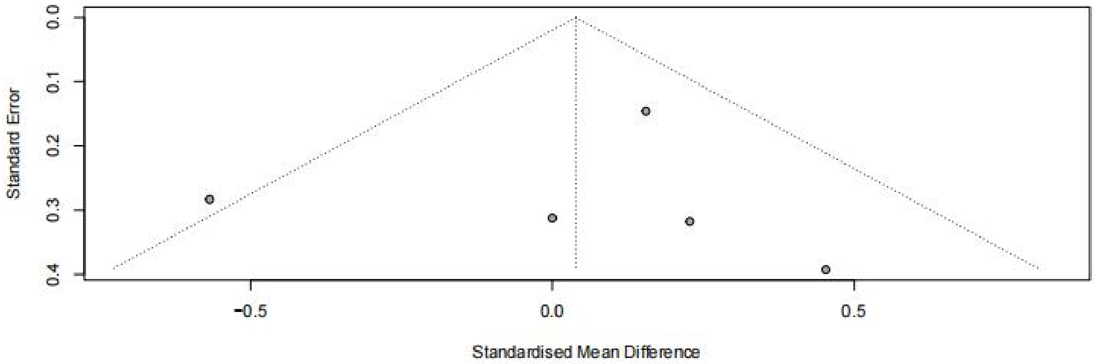

Supplement: Supplementary file 1 — Supplementary Material 1: Supplementary Appendix 1: Detailed search strategies of PubMed, Embase, Web of Science, MEDLINE and the Cochrane Library. Supplementary Table 1: Data extraction of the included studies. Supplementary Fig 1: Risk of bias of the included studies. Supplementary Fig 2. PRISMA flow chart of search and selection of studies [file 12890_2023_2782_MOESM1_ESM.pdf]
